# Herbal Theranostics: Controlled, Targeted Delivery and Imaging of Herbal Molecules

**DOI:** 10.7150/ntno.94987

**Published:** 2024-03-25

**Authors:** Aseem Setia, Bhaskar Vallamkonda, Randheer Reddy Challa, Abhishesh Kumar Mehata, Paresh Badgujar, Madaswamy S. Muthu

**Affiliations:** 1 Department of Pharmaceutical Engineering and Technology, Indian Institute of Technology (BHU), Varanasi-221005, UP, India.; 2 Department of Pharmaceutical Science, School of Applied Sciences and Humanities, VIGNAN's Foundation for Science, Technology & Research, Vadlamudi-522213, Andhra Pradesh, India.

**Keywords:** Herbal compounds, nanotheranostics, formulations, controlled and targeted drug delivery system

## Abstract

Modern medicine relies on a small number of key biologics, which can be found in nature but require further characterization and purification before they can be used. Since the herbal remedy is given through a dated and ineffective method of drug administration, its effectiveness is diminished. The novel form of medicine delivery has the potential to increase the effectiveness of herbal substances while decreasing their side effects. This is the main idea behind utilising different ways of drug delivery in herbal treatments. Several benefits arise from novel formulations of herbal compounds as compared to their conventional counterparts. These include enhanced penetrating ability into tissues, constant delivery of effective doses, and resistance to physical and chemical degradation. Controlled and targeted delivery that include herbal components allow for more traditional dosing while simultaneously increasing their efficacy. Enhancing the biodistribution and target site accumulation of systemically administered herbal medicines is the goal of nanomedicine formulations. The field of nanotheranostics has made significant advancements in the development of herbal compounds by combining diagnostic and therapeutic functions on a single nanoscale platform. It is critically important to create a theranostic nanoplatform that is derived from plants and is intrinsically "all-in-one" for single molecules. In addition to examining the mechanistic approach to nanoparticle synthesis, this review highlights the therapeutic effects of nanoscale phytochemical delivery systems. Furthermore, we have evaluated the scope for future advancements in this field, discussed several nanoparticles that have been developed recently for herbal imaging, and provided experimental evidence that supports their usage.

## 1. Introduction

A novel drug delivery system (NDDS) for herbal medicines has received a lot of focus in the last several decades. To overcome the drawbacks of conventional medication delivery methods, an NDDS technology has emerged. Nevertheless, the herbal treatment is only partially effective because the patient is given it through an antiquated and conventional drug delivery mechanism. Applying NDDS technology to herbal medicine has the potential to improve the effectiveness and decrease the adverse effects of several herbs and herbal compounds 1, 2. For herbal drugs, there are many benefits to developing nano-sized dosage forms in phytoformulation research, such as liposomes, solid lipid nanoparticles, phytosomes, nanoemulsions, polymeric nanoparticles, and nanocapsules 3, 4. These forms protect against toxicity, increase pharmacological activity, improve stability, improve tissue macrophage distribution, provide sustained delivery, and protect against physical and chemical degradation. Therefore, there may be a future for nano-sized NDDSs of herbal pharmaceuticals in improving their activity and resolving issues related to plant medicines 5, 6. Herbal medicine's role in providing primary healthcare to developing countries has unquestionably captured the interest of the global community in recent times 7. Pharmaceutical companies are hesitant to invest in drug development based on natural products and instead look into their library of synthetic molecules, even though this approach has several benefits. These compounds have unique advantages, such as reduced toxicity, low cost, and lack of side effects 8, 9. Requiring a well-validated method for component isolation and purification, low stability, and poor lipid solubility are some of the drawbacks of plant-based medicines. The challenge is entirely on the manufacturer to find a solution to these problems, so that the product is stable enough for patients to safely consume. Numerous obstacles must be surmounted to address herbal drugs. These include the following: the difficulty of performing clinical research on herbal drugs; the need for simple bioassays to standardize biological processes; the development of methods for pharmacological and toxicological evaluation; the investigation of the sites of absorption of herbal drugs; the use of toxic herbal drugs; the discovery of other animal models for toxicity and safety evaluation; and the legal and regulatory aspects of herbal drugs 10. Conventional medical practices often only allow a little concentration of the drug to reach the intended site of action. The physicochemical characteristics of the herbal products led to its widespread distribution throughout the body, which diminished its therapeutic potency 11. The instability of herbal formulations is caused by the presence of several phytoconstituents in herbal plants. For many plant species with therapeutic value, the delivery of herbal formulations to the intended place is a significant obstacle. Tannins, flavonoids, and terpenoids are examples of compounds that are water-soluble, yet have poor absorption because they cannot pass the biological membrane. They are also less efficacious and have lower bioavailability, due to their greater molecular size. Numerous advancements in targeted drug delivery, solubility, stability, bioavailability, toxicity reduction, drug molecule maintenance, and release control have been linked to the NDDS 12, 13. Nanomedicine is a rapidly developing field that aims to treat and prevent diseases by utilizing information and technology from nanoscience in remedial biology. It has also been found that nanostructures can help distribute hydrophilic medicines to their intended sites more easily and avoid drug deprivation in the intestines. Additionally, nanodrugs enhanced drug bioavailability when administered orally, likely as a consequence of the absorptive endocytosis processes they incorporated 14. There was less plasma fluctuation and fewer adverse effects because these nanostructures stayed in the bloodstream for longer and allowed medication release at a controlled rate. These structures were able to efficiently transport drugs to their intended targets because their nanoscale size allowed them to pass through cell membranes and aid in drug uptake 15. Nanoscale structures are also more efficiently absorbed by cells than bigger particles, those between 1 to 100 nm in size 16. Consequently, they were selectively effective against infected cells, leading to increased efficacy with minimal side effects. The physiochemical characteristics of nanoformulations were changed by modifying the main characteristics of nanocarriers, including their components, dimensions, forms, and surface characteristics 17. The introduction of nanoformulations is primarily intended to treat illnesses with the highest therapeutic potential and the fewest side effects. So far, the target drug's biochemical and biophysical characteristics have been the main determinants of the usage of a suitable NDDS 18.

Herbal research has been revolutionised by multifunctional preparations called "Theranostics" that combine herbal chemicals with multimodal imaging. Several people in the scientific community are interested in finding novel nanotheranostics 19, 20. The subject of theranostics nanomedicine is still in its early stages, but multimodal preparations combining phytochemicals with diagnostic agents have the potential to become NPs with magical properties and multiple uses 21-23. There is hope that next-generation particles of herbal compounds can be created through the engineering of such multi-purpose nanoplatforms for accurate diagnosis and rapid therapy. Further in-depth research is required to determine their optimal safety performance 24. Taking all of that into consideration, the purpose of this review was to detail several natural products that use NDDS, the widespread usage of these herbal compounds as nanotheranostics for a wide range of illnesses, and the many ways in which these products are prepared and used.

## 2. Rationale for developing sustained, controlled, and targeted delivery system

**Figure 1:**
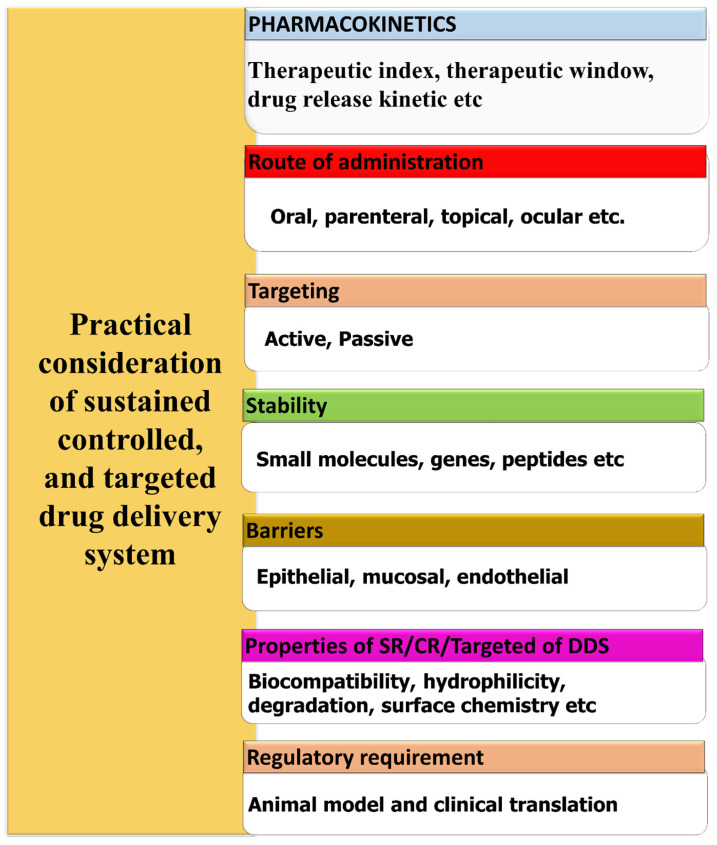
General design considerations of sustained, controlled, and targeted delivery system.

Several elements and aspects must be taken into account when planning a controlled, sustained delivery system. The design considerations are briefly shown in the Fig. 1. In general, the parameters can be categorized as either drug-related or formulation-related 25. Biomaterial characteristics, administration route, pharmacokinetics, and stability improvement are the most important formulation-related criteria. Dosage form design also heavily relies on drug-related characteristics, such as drug binding efficiency with plasma proteins, drug translocation over biological barriers, and regulatory considerations. Biocompatibility, surface chemistry, hydrophilicity, mechanical, rheological, and degradation are some of the biomaterial qualities that require investigation 26. It is also necessary to evaluate the biomaterials' behavior in different pH and temperature ranges. To select an appropriate biomaterial and develop an appropriate dosage form, knowledge about the drug administration routes is essential. When developing a controlled release carrier, it is important to take stability into account for pharmaceuticals. One way to accomplish this is by using specialized carrier systems that incorporate specific medications 27. To avoid toxic side effects in other parts of the body, it is crucial to direct the medication just to the area where it will have the desired pharmacological effect. Certain organs, such as the brain, bone, and testicles, are difficult to target for medication delivery due to biological barriers 28. As an alternative, drugs that have been engineered with permeation enhancers and nanocarriers can penetrate these barriers and reach their intended target site. To achieve the optimal *in vitro in vivo* correlation (IVIVC), it is necessary to develop appropriate animal models for every type of delivery mechanism. The United States Food and Drug Administration (FDA) defines an IVIVC as a mathematical model that predicts the link between an oral dosage forms *in vitro* characteristic and relevant *in vivo* response 29.

## 3. Approaches for developing herbal drug delivery systems

**Figure 2:**
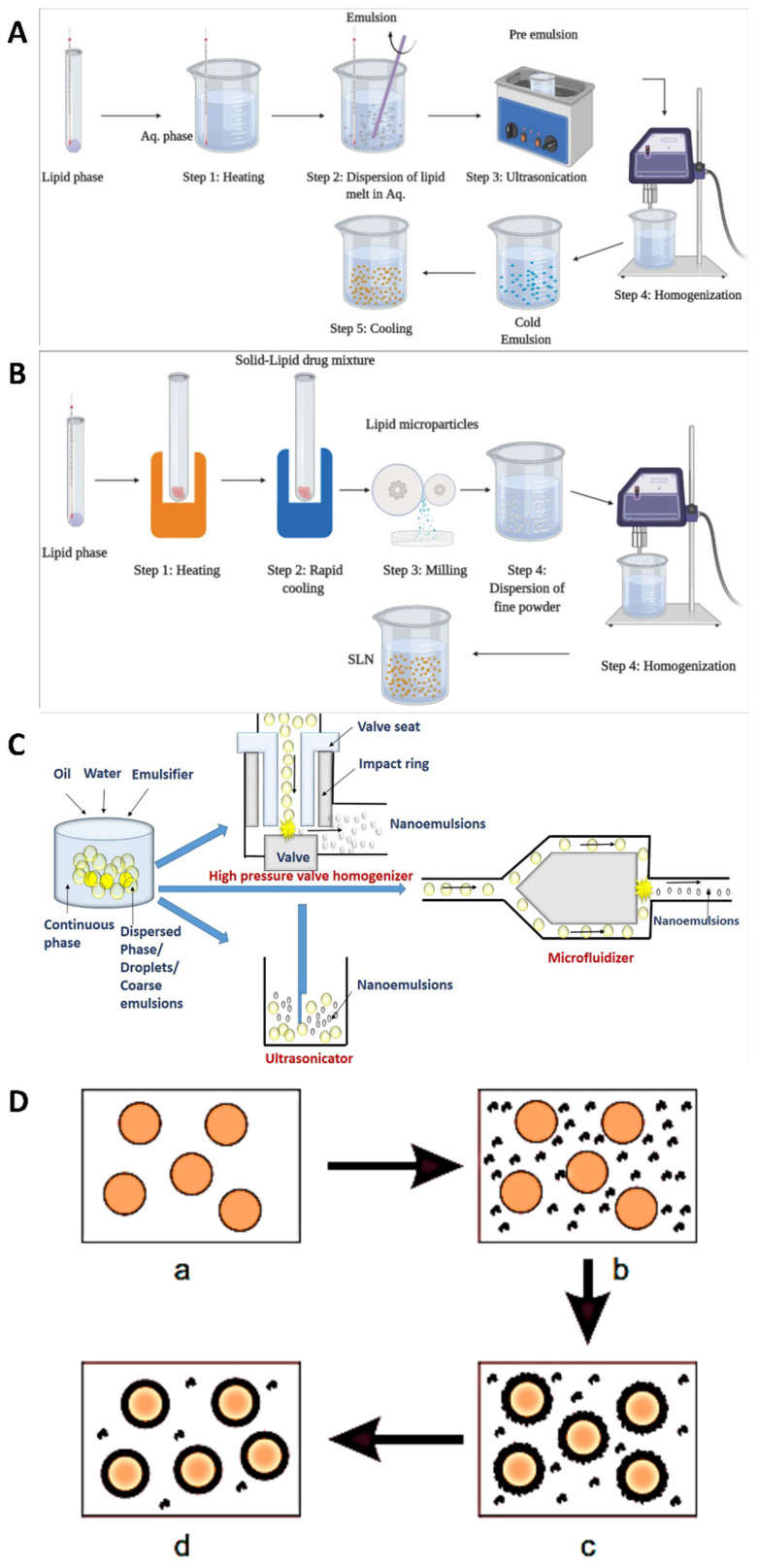
Mechanistical approach for the preparation of nanoparticles (A) Hot homogenization; (B) Cold homogenization Reproduced with the permission ref 65. Fig. 3 and Fig. 4. (Open Science Publishers) (C) High pressure homogenization. Reproduced with the permission ref 66. Fig. 2 (Frontiers); (D) Coacervation technique. Reproduced with the permission ref. 67. Fig. 1 (MDPI).

Various techniques are utilised in the development of modified drug delivery systems to boost medicine effectiveness, lessen adverse effects, and enhance patient compliance. When it comes to designing customised drug delivery systems, these approaches cover a wide spectrum of strategies 30. To improve drug bioavailability and the proportion of drug deposited in the desired zone, decrease drug degradation and loss, and avoid adverse side effects, several drug targeting and delivery methods are presently being developed. To tailor the carriers to a specific area of interest, they can be engineered to degrade slowly, react to certain stimuli (such as changes in pH or temperature), or even be targeted 31. Pharmaceuticals that rely on natural substances found in plants rather than synthetic ones are known as phytopharmaceuticals. The body processes and uses natural nutrients more efficiently. Consequently, they are more effectively absorbed into the bloodstream and cause fewer side effects. Side effects are a common occurrence with pharmaceuticals manufactured from chemical substances. Some synthetic chemical substances will be spontaneously rejected by the human body. Recently, tissue-engineered skin has become a reality. Obtaining barrier function through laboratory multiplication of skin cells can be a life-or-death decision for individuals suffering from significant full-thickness burns 32. These rejections manifest as side effects, ranging from relatively harmless ones like headaches to more serious ones that could be fatal. While phytopharmaceuticals typically have little, if any, negative side effects, it's nevertheless possible for them to interact chemically with other medications. In addition, unlike botanicals, these chemicals are single and purified, so they may be easily standardised and used in modern drug delivery systems 33. The mechanistical approach for the preparation of nanoparticles are shown in Fig. 2. The complete descriptions of different nanocarriers are shown in Table 1.

### 3.1. Method of hot homogenization

Nanoparticle formulations, such as medication delivery systems or nano emulsions, are often prepared by hot homogenization. The application of heat is important to this process, which enables the formation of stable nanoparticles. Depending on the materials, nanoparticle qualities required, and the application, the details of the hot homogenization process can differ 51. However, the basic principles are the same across all formulations. When materials are exposed to high temperatures for a short period, they undergo hot homogenization, which shrinks particles because the inner phase viscosity is lowered. This property is particularly useful for drugs that are sensitive to certain levels of heat. The combination of an emulsifier and the small particle size leads the medication and the carrier to degrade faster at higher temperatures. For hydrophilic drug candidates, hot homogenization is not the best method because it causes burst release, since the drug is partitioned into the watery process during homogenization, and many of the drug particles remain on the solid lipid nanoparticles (SLNs) outer surface when cooled 52. A heated aqueous surfactant solution is mixed with the drug-containing melt in the hot homogenization method. Following homogenization using a piston-gap homogenizer, the heated O/W nano emulsion is cooled to room temperature, resulting in the production of stable lipid nanoparticles 53.

To homogenize an emulsion, it is necessary to use temperatures greater than the lipid's melting point. When combining lipids and drugs at the same temperature, an aqueous surfactant is employed. An oil-in-water emulsion is created by heating a pre-emulsion using a high-shear mixing apparatus. After that, the product is cooled, which triggers the creation of lipid crystals and SLNs 54. Three to five homogenization cycles at 500-1,500 bar are required to produce flawless SLNs. One must constantly keep in mind that high pressure causes a rise in temperature. The size of the particles increases, as the pressure or number of cycles increases. This is because the energy required to move the particles creates attractive interactions between them. Lastly, the nanoemulsion is cooled to room temperature. This causes the lipids to recrystallize, resulting in the production of nanoparticles 55.

In a study, Kumar et al., formulated SLNs of curcumin. This research set out to find a way to make curcumin SLNs that would have a better pharmacological activity and a higher bioavailability profile. A hot homogenization coupled with an ultrasonication method was used to make the SLNs. Poly vinyl alcohol, tween 80, and tripalmitin were the ingredients to produce SLNs. After optimising the blank SLNs formulations, they were tested for drug encapsulation effectiveness, shape, zeta potential, particle size, and *in vitro* drug release. The curcumin was then encapsulated using these formulations. To verify the drug-lipid-surfactant cross-linking reaction, FT-IR spectroscopy was used to examine the produced SLNs. The SLNs had a particle size of 214.60 ± 3.55 nm, a polydispersity index of 0.49±0.03, a zeta potential of -29.63 ± 0.50 mV, an encapsulation efficiency of 51.99 ± 4.14%, and a loading capacity of 5.33 ± 0.34%. An initial burst release of 16.5% within 2 hours and subsequent continuous release over 96 hours were the two phases of the biphasic pattern observed in *in vitro* drug release. The FTIR analysis revealed that the lipid and surfactant mixture was the only thing that occurred during the formulation process; the medication and the surfactants did not react to produce any reactive products 56.

### 3.2. Techniques for cold homogenization

To achieve uniform dispersion of nanoparticles in different matrices, which is essential for their optimal performance. A cold homogenization procedure that disperses nanoparticles in a matrix at low temperatures to avoid agglomeration while preserving their desirable properties is one efficient method 57. The complexity of the nanoemulsion's crystallisation step causes multiple modifications and/or super cooled melts; temperature-induced drug degradation; drug distribution into the aqueous phase during homogenization; and these are just a few of the issues that cold homogenization aims to address. This method involves fast cooling the drug using cryogenic devices such as liquid nitrogen or ice nitrogen, after it has melted in the lipid melt. Afterward, blend it into a fine powder using a powder mill. After that, to obtain a nanoparticle, homogenize at or below room temperature 58.

Ahangarpour et al., examine the impact of myricitrin SLNs on hyperglycemic myotube and streptozotocin-nicotinamide-induced type 2 diabetes in mice. The SLNs utilised in this experiment was prepared using the cold homogenization procedure. Then, 120 adult male NMRI mice were split into seven groups: control, vehicle, diabetes (myricitrin 1, 3, and 10 mg/kg added to SLNs), diabetes (myricitrin 65 mg/kg given 15 minutes after NA 120 mg/kg injection), and diabetes (myricitrin + metformin). The *in vitro* investigation utilised these cell lines for analysis. Experimental evaluations were conducted on plasma samples, pancreatic and muscle tissues, and myotubes following the final nanoparticle treatment. Hyperglycemia, insulin resistance, and pancreatic apoptosis are outcomes of diabetes, which also increase lipid peroxidation and decrease antioxidant defense. Cellular death, antioxidant deficiency, and oxidative stress were all brought about by hyperglycemia. In both animal and laboratory tests, myricitrin SLNs reduced the risk of diabetes and hyperglycemia consequences. Consequently, myricitrin SLNs has anti-apoptotic, antioxidant, and antidiabetic properties in both myotube cells and mice 44.

### 3.3. Methods of homogenization under high pressures

For the processing of lipid-based nanoparticles, polymeric nanoparticles, and inorganic/hybrid-type nanoparticles, high pressure homogenization (HPH) has become a practical method. A growing number of therapeutically relevant nanoparticle processing steps now include HPH. Research into the HPH process has focused on nanoparticle manufacturing to better understand the process and draw conclusions about how to optimise, manage, and scale it up 59. Producing SLNs in a continuous process at elevated temperatures and pressures, the hot melt extrusion approach ensures that the final products have consistent densities, morphologies, and forms. Hot melt extrusion and high-pressure hydrolysis are two processes, when combined it allow the pharmaceutical industry to manufacture SLNs formulations with reduced particle sizes 60. The two procedures could be combined to create a scalable method for SLNs. First, the raw materials could be pumped into the extruder barrel at a temperature higher than the lipids' melting point. Second, the SLNs could be made smaller by attaching a high-pressure homogenizer to the end of the hot melt extruder barrel through an insulated connector. In comparison to the standard method, the aforementioned procedure yielded SLNs with superior size reduction and process parameters. The most important process parameters that affect the SLNs size are the lipid concentration, screw design, and residence duration 61.

Sun et al., developed curcumin loaded SLNs (C-SLNs) using high-pressure homogenization. Researchers looked at the improved formulation's curcumin shape, stability, and release. The MCF-7 cells were used to assess the formulation's anti-cancer efficacy. The drug's uptake by cells was measured using fluorescence spectrophotometry. After being administered intravenously, the pharmacokinetic characteristics of curcumin in SLNs were examined in rats. The results showed that whilst the drug loading was not improved, the particle size was lowered by blending Sefsol-218(®) into a lipid matrix. An optimised mixture was made containing 0.8% drug, consisting of 630:70:300 w/w of Dynasan 114(®), Sefsol-218(®), and Pluronic F68(®). The dispersion of this formulation in water was achieved with an entrapment efficiency of 90% and an average particle size of 152.8 ± 4.7 nm. Improved chemical stability was demonstrated by curcumin's two-phase sustained release profile from C-SLNs. There was a time-dependent increase in intracellular absorption and C-SLNs showed extended inhibitory effect in cancer cells compared to the solubilized solution. The bioavailability of curcumin was enhanced 1.25-fold following intravenous dosing to rats. C-SLNs that are more chemically stable and easier to disperse in water have been created. C-SLNs have the makings of a promising curcumin delivery method for cancer treatment 45.

### 3.4. Method for complex coacervations

According to Burwell (1976), a colloidal solution undergoes coacervation when it splits into two liquid phases. A single polymer can be coacervated by adding a salt or a dissolving agent; examples of such agents include alcohol and acetone. The process of complicated coacervation causes an electrostatic interaction between two or more polymer solutions in water that have opposite charges. This interaction creates two immiscible liquid phases: one that is dense with polymers and another that is low in polymers 62. The former is a covering for many different types of core materials; it is sometimes referred to as coacervate. Several phases are often involved in the complex coacervation process. The core components are first dissolved in a water solution of cationic polymers, typically a protein. The next step is to incorporate a carbohydrate-based anionic polymer solution. Coacervate microdroplets begin to dissociate from the continuous polymer phase as a result of temperature and pH adjustments. Coacervate microdroplets respond to water-insoluble core materials by interacting with their surfaces and eventually forming a continuous shell. To make the gel stronger, crosslinking agents like glutaraldehyde and transglutaminase are commonly utilised. Many substances, including fatty acids, fat-soluble vitamins, and flavours, are insoluble in water and can be treated using the complicated coacervation approach 63.

Amani et al., developed curcumin loaded tragacanth and gelatine (GE) film with coacervation technique. Different ratios of gelatin to a soluble fraction of tragacanth gum (SFTG) were used to generate curcumin active films in this investigation. The ratios were 1:1 and 2:1 using complicated coacervation. The mechanical characteristics, thickness, and water vapour percentage (WVP) of the finished films were unaffected by the different biopolymer ratios used. The release rate, swelling ratio, water solubility, and moisture content were all affected by the biopolymers' ratio. Films containing 1GE:1SFTG had a decrease in tensile strength (from 1.74 MPa to 0.62 MPa) and 2GE:1SFTG films had an increase in elongation at break (from 81.48% to 122.00% and 98.87% to 109.58% MPa, respectively), when curcumin was blended with biopolymers. Adding curcumin to films reduced their moisture content and water solubility. Films infused with curcumin exhibited an antioxidant activity, that was about five times more than that of untreated film samples. In addition, FTIR analysis confirmed that an amide linkage had been created by the interaction of the carboxylic group of SFTG with amide I of GE. When comparing the film samples to the primary constituents, TGA revealed a decrease in thermal stability 64.

## 4. Novel drug delivery systems (NDDS) based herbal actives

### 4.1. Solid Lipid Nanoparticle (SLNs) of herbal bioactives a NDDS

In the early 1990s, researchers created SLNs, a colloidal drug carrier with particles ranging in size from 50 to 1000 nm. To formulate SLNs, a mixture of melted solid lipids in water is stabilised by adding an emulsifier 68. Micro emulsification and the high-pressure homogenization technique are the two most used ways to make SLNs. The main advantages of SLNs are its lipophilic lipid matrix for drug dispersion and its ability to help transport therapeutic loads to targeted cells and tissues 69. Additionally, SLNs allows for the encapsulation and embedding of a wide range of molecules, including drugs, antigens, proteins, and nucleotides. One of the desirable aspects of SLNs is its capacity to improve stability both *in vitro* and *in vivo* while simultaneously decreasing side effects. SLNs and nanoemulsions are quite similar; the main difference is that SLNs uses solid and liquid lipids (oils) in its formulation, while nanoemulsions solely employ liquid lipids 70.

Aanisah et al., prepared SLNs-based hydrogel for safflower petals extract (SPE) to improve the solubility and penetration of two bioactive chemicals identified in SPE. The SLNs were synthesised using the hot emulsification-ultrasonication technique, and the extraction process included 100% v/v ethanol to achieve strong antioxidant activity. The method was able to capture more than 80% of QU and LU was demonstrated by the results. Additionally, FTIR, DSC, and PXRD spectra showed that the majority of the QU and LU were embedded in a lipid matrix and distributed uniformly at the molecular level, which enhanced their solubility. The SLNs-hydrogel composites not only showed a 19-fold increase in skin retention and LU and QU penetrability, but they could also release two lipophilic bioactive chemicals over 24 hours. Below 500 μg/mL, there was no evidence of hemolytic toxicity, according to *in vitro* blood biocompatibility. The formulation was thus deemed to be free of any known safety concerns. Results above 15 on the sun protection factor (SPF) test indicate a very promising photoprotective agent for preventing signs of photoinduced skin ageing 71.

**Figure 3:**
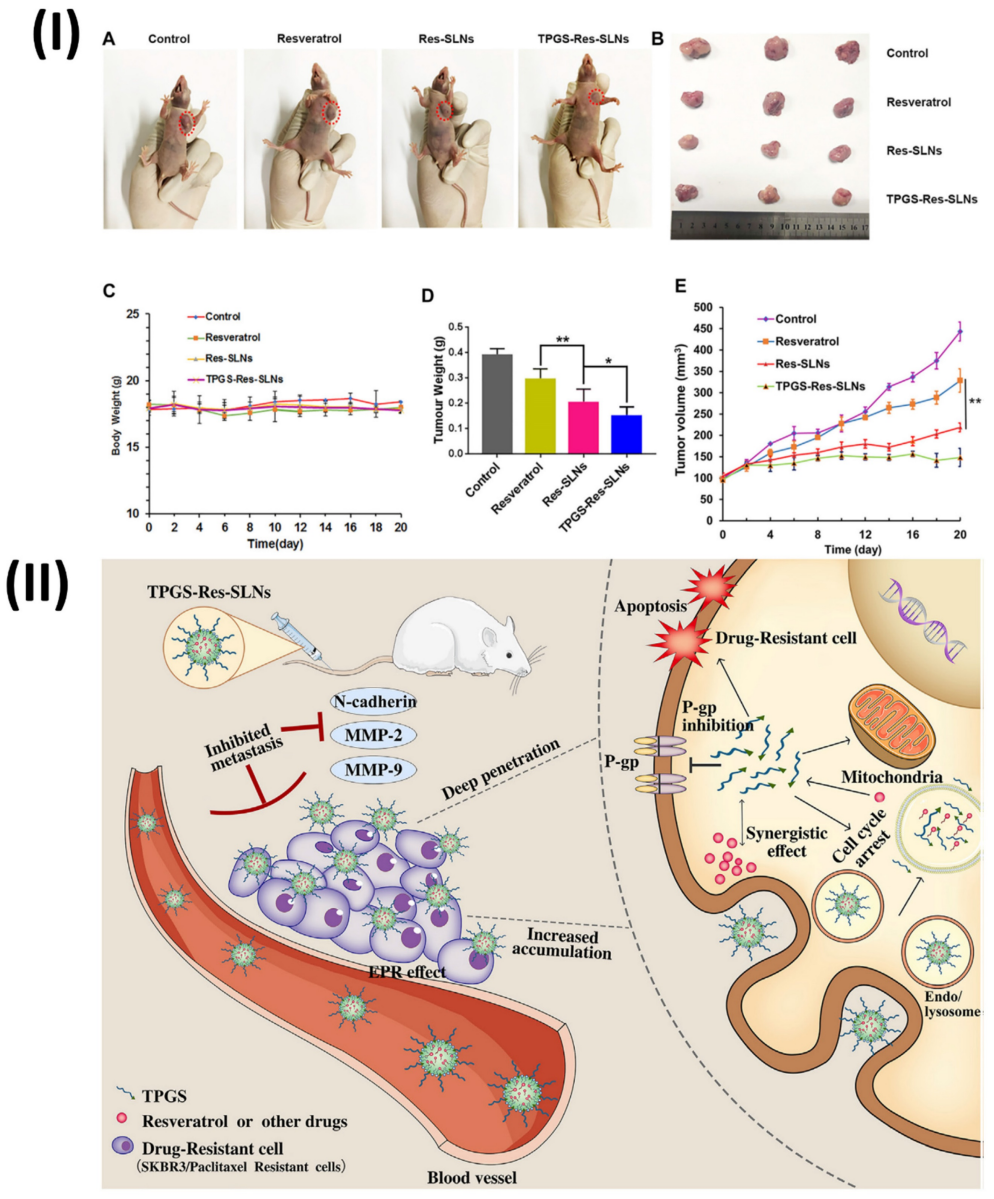
(I) Results of Resveratrol, Res-SLNs, and TPGS-Res-SLNs administered to mice with SKBR3/PR xenografts when administered in vivo; (A) Mice from each treatment group on day 16 as shown in the representative photographs. (B) Digital photos of tumours removed from individual mice after the relevant treatments. (C) Mice given the specified formulas showed a weight-versus-time curve. (D) Mouse tumour mass measured across all treatments. (E) Mice treated with the specified formulas showed tumour volume versus time graphs; (II) The process by which TPGS-Res-SLNs control paclitaxel resistance in breast cancer, as shown schematically. Reproduced with the permission from ref. 72. Fig. 7, and Fig. 9 (Frontiers).

Wang et al., formulated TPGS resveratrol solid lipid nanoparticles (TPGS-Res-SLNs) for breast cancer therapy. The TPGS-Res-SLNs were prepared using the solvent injection approach in this investigation. The TPGS-Res-SLNs showed a zeta potential of -25.6 ± 1.3 mV and a drug loading of 32.4 ± 2.6%, respectively. The TPGS-Res-SLNs' ability to cause mitochondrial malfunction, boost tumour treatment efficacy through apoptosis, and enhance cellular uptake of chemotherapeutic medicines was thus clearly demonstrated. Also, TPGS-Res-SLNs significantly slowed down cell migration and invasion in SKBR3/PR cells compared to free resveratrol. Additionally, in vivo SKBR3/PR xenograft tumour models showed that TPGS-Res-SLNs promote tumour cell death more effectively than free resveratrol, leading to high therapeutic outcomes on tumours. Ultimately, this study's results point to TPGS-Res-SLNs' promising future as a drug delivery vehicle for breast cancer treatment, specifically in the fight against drug resistance (Fig. 3) 72.

### 4.2. Nanostructured Lipid Carrier (NLC) of herbal bioactives a NDDS

Originating from SLNs with an increased number of lipid matrix defects, it is now thought of as a second-generation lipid nanoparticle containing a combination of solid and liquid lipids. In contrast to the most popular liquid lipids—olive, mustard, castor, and cod liver oil a large range of solid lipids including hydrogenated palm oil (HPO), glyceryl monostearate, stearic acid, and cetyl alcohol have been employed. Thimerosal is the best stabilizer to use in this system 73. In comparison to SLNs, NLC offers several advantages, such as improved stability, more drug-loading capacity, less drug ejection during storage, and greater control of drug release. Therefore, NLC has been formulated with many active ingredients to study how they affect water solubility, absorption in the gastrointestinal tract and oral bioavailability, release control, circulation time extension through reduced reticuloendothelial system (RES) identification, and co-delivery. The superiority of NLC as a vehicle for the oral administration of several naturally occurring and synthetically produced substances has been thus established 74. Lacatusu et al., formulated hydrogels (HG) with nanostructured herbal ingredients that can improve the transdermal absorption of bioactive chemicals derived from specific plant extracts and oils. The discovery of prototype items that produce an enhanced therapeutic response through a combination of antioxidant, anti-inflammatory, and antiacne effects. There was a strong capacity to capture both short- and long-life free radicals, as well as a high level of biocompatibility when Carrot Extract (CE) and Marigold Extract (ME) NLC based on rosehip oil or black cumin oils were combined. *In vitro* studies have demonstrated that HG-NLC-ME-CE is a capable carrier with a differentiated capacity for the release of the two active principles; for instance, AA, a hydrophilic active, was released more quickly than carotenoids. The decrease in inflammatory cytokine expression (IL-1β and TNF-α), with TNF-α showing the most significant effect, was shown by the *in vitro* and *in vivo* studies that confirmed the efficacy of HG-NLC in treating skin inflammation. It was also found that HG-based NLC-CE/ME-AA had a better anti-inflammatory effect *in vivo* than a commercial product; specifically, a substantial decrease in rat paw oedema was assessed after 3 hours of treatment with HG-NLC. The created prototypes showed great promise in pre-clinical testing, with an assessment of hydration and elasticity effects in living epidermis providing evidence of their suitability for commercial deployment. Applying the created prototypes topically significantly improved the skin's hydration and elasticity; researchers measured an increase in hydration of up to 74% and an increase in elasticity of up to 90%. To achieve better therapeutic efficacy and no drug toxicity, the cosmetic industry could use the knowledge acquired from this investigation to create new topical products with health benefits, antioxidants, anti-inflammatory, and anti-acne properties, as well as the hydration and elasticity profiles desired 75.

**Figure 4:**
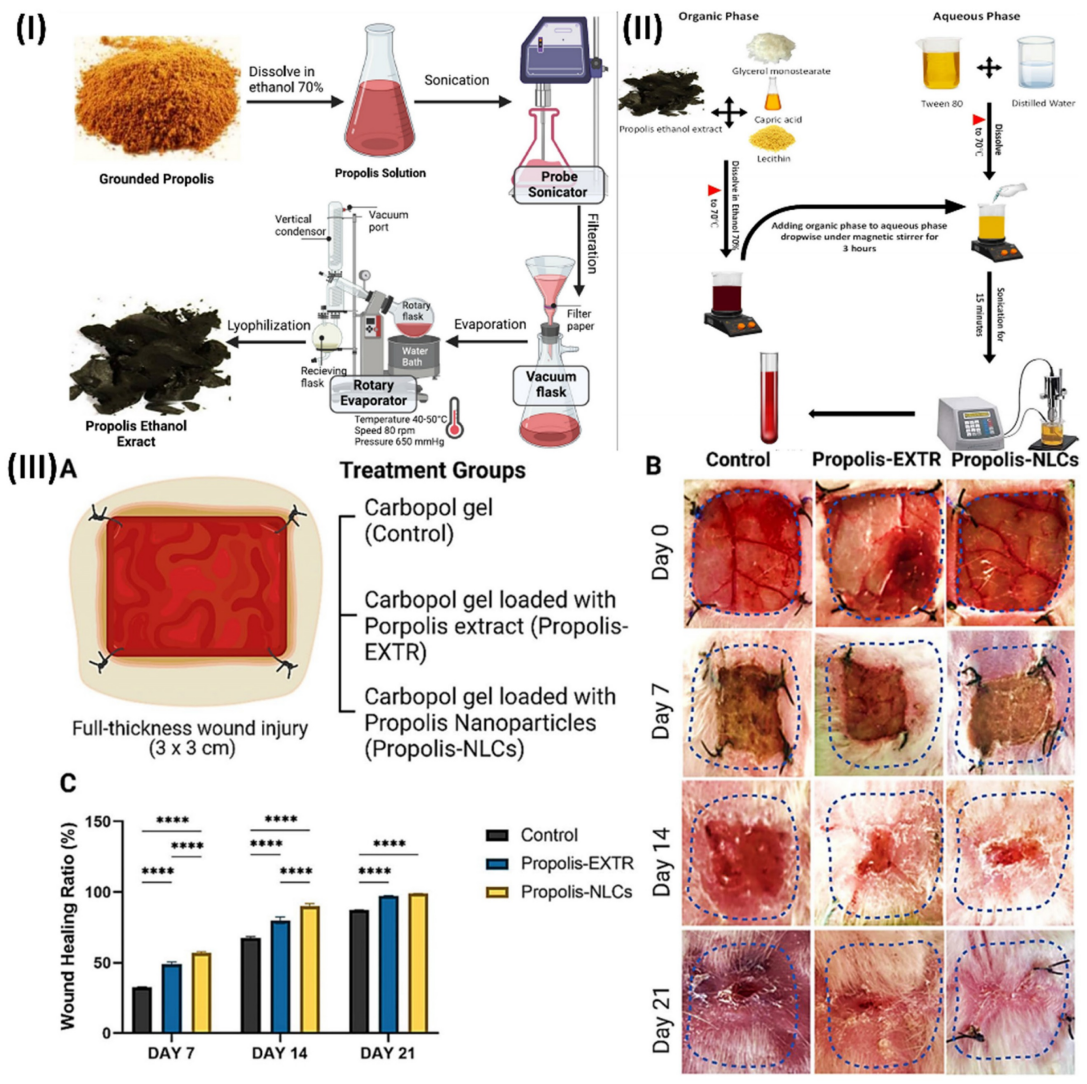
(I) Extraction of raw, ground propolis using hydro-alcoholic solvents; (II) Development of Propolis-Based NLCs; (III) Evaluation of the efficacy of Propolis-EXTR and Propolis-NLCs in promoting wound healing in contrast to a control group administered carbopol alone. (A) A schematic depicting the experimental design for a rabbit study including a full-thickness skin damage and the treatment groups. (B) The sizes of wounds are shown in representative photographs. (C) Ratio of wound healing in the control, Propolis-EXTR, and Propolis-NLCs groups following injury induction (0 day), 7, 14, and 21 days later for qualitative analysis. Reproduced with the permission from ref. 76. Fig. 1, Fig. 2 and Fig. 6 (Springer).

Elkhateeb et al., inspected the potential benefits of incorporating nanotechnology into the herbal extract (propolis rebuts), and then they tested its ability to promote wound healing. First, three quantities of propolis NLCs were produced utilising the emulsion-evaporation-solidification process. Their phenolic and flavonoid contents were subsequently compared. They next examined the antioxidant, antibacterial, and antifungal activities of propolis-NLCs. Finally, they used rabbits with full-thickness skin wounds to evaluate their skin regenerating capacity. When contrasted with raw propolis extract (EXTR), the phenolic and flavonoid contents of propolis-NLCs were found to be nine times and two times higher, respectively. Antioxidant activities were 25 times greater than those of propolis-EXTR. In terms of inhibitory activity on Gram-positive (Bacillus subtilis and Staphylococcus aureus), Gram-negative (Salmonella spp.), and fungal (Candida albicans) microorganisms, propolis-NLCs were twice as effective as propolis-EXTR. After two weeks, the wounds in full-thickness skin injuries treated with propolis-NLCs were much more closed than those in propolis-EXTR and the control group. In comparison to propolis-EXTR, propolis-NLCs showed greater skin regeneration power and a noticeable broad-spectrum antibacterial impact. In addition, they suggested nanotechnology as a possible treatment for wound healing by highlighting its additive effect on herbal extract, which improved its antioxidant and antibacterial properties due to the extract's higher flavonoid content (Fig. 4) 76.

### 4.3. Niosome of herbal bioactives a NDDS

The lamellar phase of nonionic amphiphilic lipids encases a watery core in a nonionic nanosphere vesicle called a niosome, which can have a diameter ranging from 100 nm to 2 µm 77. Niosomes are structurally very similar to liposomes, however, they have the potential to give greater benefits than liposomes, due to their increased penetration capabilities, stability, and therapeutic index of a medicine, as well as their decreased toxicity. Among niosome's many benefits are its low price, great solubility, adaptability, and controlled release of its contents. Transdermal delivery, peptide transport, haemoglobin delivery, and neoplasia targeting have all made extensive use of them 78.

Raafat et al., prepared Fumaria officinalis noisome for antineuropathic and anti-inflammatory potentials. Finding the primary active components of Fumaria officinalis is the primary goal of this bio-guided phytochemical inquiry. Acute, subchronic, and chronic diabetic neuropathy and diabetes caused by alloxan, as well as acute inflammatory pain and chronic inflammatory edema caused by carrageenan, were investigated in an *in vivo* biological evaluation. The two main alkaloids found in alkaloid-rich fraction (ARF) were stylopine (48.3% concentration) and sanguinarine (51.6%). Optimal niosomal formulations were determined by in-vitro optimisation, analytical, and *in vivo* biological research. Nio-2 was the most optimised of these formulations. The pharmacokinetic parameters of ARF were improved by Nio-2, which had a particle size of 96.56 ± 1.87 nm. Perhaps the primary mechanism by which they exert their antinociceptive and anti-inflammatory effects is by decreasing levels of the pro-inflammatory factor's, tumour necrosis factor-alpha (TNF-alpha) and interleukin 6 (IL-6), increasing levels of the anti-inflammatory factor IL-10, and ameliorating the *in vivo* oxidative stress. This study presents a new and practical oral formulation that effectively alleviates neuropathic pain, as well as other inflammatory disorders and consequences of diabetes 79.

**Figure 5:**
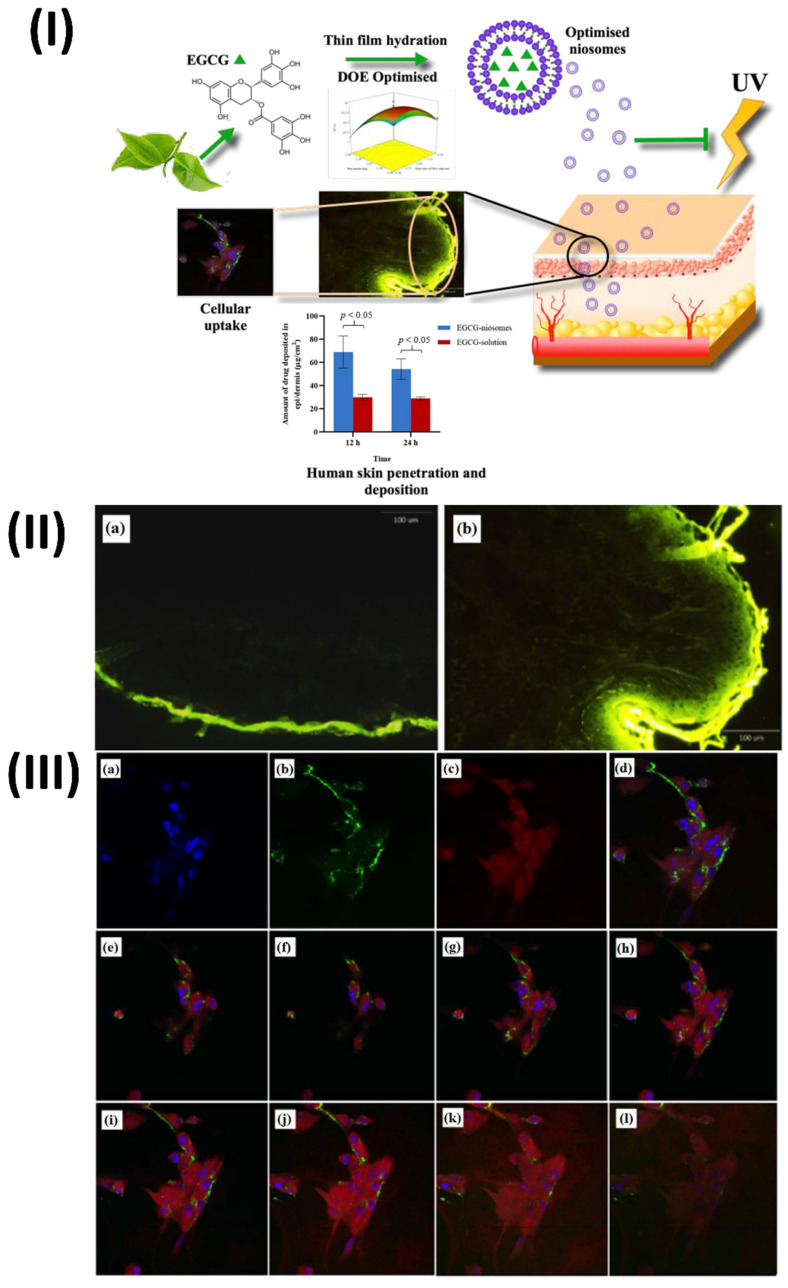
(I) Technique of niosomes preparation and cellular mechanism (II) Sections of human skin treated with a FITC solution show the whole thickness of the skin. (III) Perinuclear particle accumulation is shown in confocal laser scanning microscopy pictures of Fbs following 2 hours of incubation with FITC-labelled nanosomes at 37 °C. Reproduced with the permission from ref. 80 Graphical abstract, Fig. 6, and Fig. 9 (MDPI).

Li et al., develop and statistically optimise an EGCG loaded niosomal system. EGCG-niosomes were optimized statistically after being made using the thin film hydration process. Measurements of niosome size, zeta potential, shape, and entrapment effectiveness were taken. Studies on penetration and deposition were carried out using human skin that was fully thickened. They measured cellular absorption, antioxidant enzyme activity following UVA-irradiation, lipid peroxidation, and cell survival. The optimized niosomes were round and of a consistent size, measuring 235.4 ± 15.64 nm. They possessed a zeta potential of 45.2 ± 0.03 mV and an EE of 53.05 ± 4.46%. Skin penetration and deposition were significantly enhanced by the niosomes compared to free EGCG, and the drug release was successfully delayed. Compared to free EGCG, they improved cell survival after UVA-irradiation, decreased lipid peroxidation, and boosted the activities of antioxidant enzymes in human dermal fibroblasts (Fbs). The niosome uptake occurred through energy-dependent endocytosis. Pharmaceutical and cosmetic companies may employ the improved niosomes as a skin transporter for antioxidants and other medicinal chemicals (Fig. 5) 80.

### 4.4. Liposomes of herbal bioactive a NDDS

**Figure 6:**
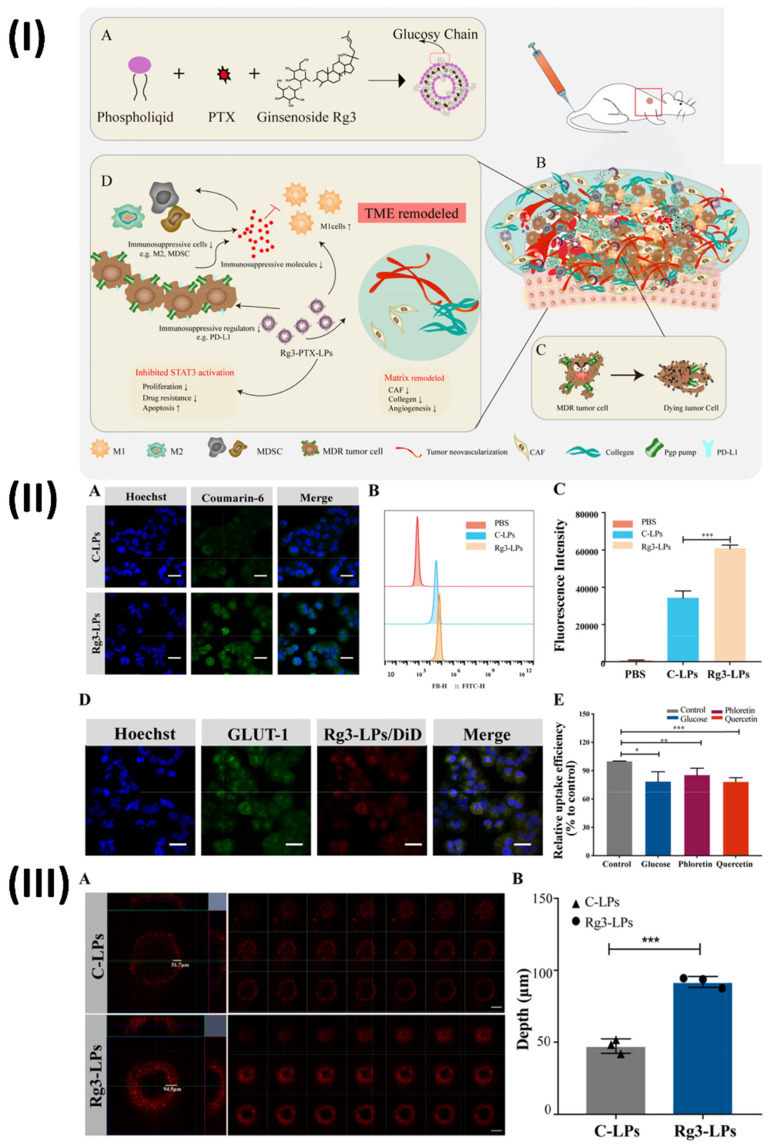
(I) A diagram depicting the Rg3-PTX-LPs system for overcoming resistance to cancer drugs; (IIExamining the cellular absorption of C-LPs and Rg3-LPs labelled with courmarine-6 in MCF-7/T cells using fluorescence microscopy. (A) Confocal fluorescence microscopy of C-LP-and Rg3-treated MCF-7/T cells. (B) the liposomes' cellular uptake was examined using FACS. (C) Efficiency of cellular absorption of C-LPs and Rg3-LPs. (D) Relationship between Rg3-LPs and MCF-7/T cells in vitro. The location of GLUT-1 and representative fluorescence pictures of Rg3-LPs in MCF-7/T cells. (E) Flow cytometry-based cellular absorption of Rg3-LPs in the presence of GLUT inhibitors compared to the control; (III) C-LPs and Rg3-LP accumulation within tumours in vitro using 3D MCF-7/T cell tumour spheroids. (A) Confocal microscopy applied to DiD for fluorescence analysis. The number of C-LPs and Rg3-LPs that accumulate determines the intensity of the fluorescence. Compared to C-LPs, Rg3-LPs showed a stronger fluorescence signal. (B) Quantitative evaluation of C-LP and Rg3-LP penetration depth in MCF-7/T spheroids. Reproduced with the permission from ref. 86. Scheme 1, Fig. 2, and Fig. 3 (Elsevier).

Liposomes are biodegradable and can improve paracellular and transcellular drug transport, making them a promising delivery strategy for herbal extracts. Liposomes are phospholipid-based delivery vehicles that encapsulate hydrophilic and lipophilic components. They self-assemble and mimic cell function. Two groups, one polar and one non-polar, define liposomes 81. Based on their membrane structure, they can be categorised as either multi-lamellar (≥400 nm), with concentric phospholipid layers, or uni-lamellar (<20 nm), with a single phospholipid bilayer encircling the aqueous phase. According to their intended use, liposomal systems can be categorised into four distinct generations: conventional, stimulus-responsive, stealth, ligand targeted, and theranostic (including newer types like transfersomes and niosomes) 82. In addition to their imaging capabilities, these liposomes have therapeutic potential. Controlled release, site-specific targeting, and biomarker capabilities allow therapeutic components to react to both internal and exterior stimuli. Theranostic liposomes are formed by adding therapeutic medicines to either the hydrophilic core or the lipophilic bilayer 83. On the other hand, diagnostic chemicals can be enclosed within the hydrophobic core or covalently conjugated on the surface of the liposome. Notable features of these liposomes include controlled release and extended circulation times 84. Ochi et al., used HepG2 cell line to assess the efficacy of a co-encapsulated pegylated nano-liposome system that contained the herbal anti-tumor medications silibinin and glycyrrhizic acid. In this investigation, nano-liposomes were co-encapsulated using sonication at 60% amplitude and a thin-layer film hydration technique with a HEPES buffer. A specific molar ratio of DPPC, cholesterol, and mPEG2000-DSPE was used to generate liposomes. Over 48 hours, they applied the MTT test to HepG2 and fibroblast cell lines to determine the cytotoxicity of different doses of co-encapsulated nano-liposomes, free silibinin (25% w/v), and glycyrrhizic acid (75% w/v). A limited size distribution was observed with an average diameter of 46.3 nm in the formulation of pegylated nano-liposomes. While glycyrrhizic acid had an encapsulation efficiency (EE) of 68.78%, silibinin only managed 24.37%. There were far more co-encapsulated nano-liposomes on the HepG2 cell line as reported in the *in vitro* cytotoxicity results when contrasted with the fibroblast cell line. The HepG2 cell line showed an IC_50_ value of 48.68 µg/ml for co-encapsulated pegylated nanoliposomal herbal medicines and 485.45 µg/ml for free silibinin with glycyrrhizic acid. The results of this *in vitro* investigation showed that silibinin and glycyrrhizic acid, when enclosed in nano-liposomes, enhanced the biological activity of free medicines, made silibinin more stable, and enhanced the therapeutic efficacy of both compounds. Compared to the combination of free silibinin and glycyrrhizic acid, the IC_50_ of the co-encapsulated nano-liposomes was lower on the HepG2 cell line 85. In another study, Zhu et al., developed ginsenoside Rg3 liposomes loaded with paclitaxel for the treatment of cancer cells and tumour microenvironment that are resistant to cancer drugs Ginger root extracts Using the thin-film hydration approach, Rg3-PTX-LPs were created by formulating unique Rg3-based liposomes loaded with PTX. Particle size analysis using dynamic light scattering was used to assess the stability of the Rg3-PTX-LPs. Using an in vivo imaging model, they investigated the active targeting effect of liposomes based on Rg3 in an MCF-7/T xenograft system. In order to assess the anticancer activity and mechanism of Rg3-PTX-LP, several in vitro and in vivo experiments were conducted on MCF-7/T cells, including MTT, apoptotic tests, TAM modulation, and TME remodelling. The results showed that Rg3-PTX-LPs were able to target MCF7/T cancer cells and the TME all at once, mostly by recognising GLUT-1. When contrasted with traditional cholesterol liposomes, Rg3-PTX-LPs demonstrated far superior drug resistance reversal capability and in vivo anticancer efficacy. Thus, Rg3-PTX-LPs attained a high tumour suppression rate of 90.3% by simultaneously targeting tumour cells and remodelling the TME (Fig. 6.) 86.

## 5. Nanotheranostics for multiple disease of herbal bioactives

The field of nanotheranostics, which involves combining nanoscale diagnostics and therapy, shows great potential for treating a variety of ailments with bioactive herbs. Personalized treatment plans are made possible by this novel strategy, which combines diagnostic capabilities with focused delivery of medicinal drugs 87. The medicinal potential of herbal bioactives is being more acknowledged due to their wide range of pharmacological actions and their natural origins. Researchers have found that herbal bioactives can be made more effective in targeting, have better stability, and are more bioavailable when combined with nanotheranostic platforms. By choosing bioactives with appropriate pharmacological characteristics, these platforms can be used to treat particular diseases, including cancer, cardiovascular disorders, and inflammatory ailments 88. There are several benefits to using herbal bioactives in nanotheranostics, such as decreased toxicity, increased efficacy, and the possibility of bioactive synergy. Herbal chemicals are also well-suited for use in long-term therapy since they are biocompatible. In addition, disorders affecting inaccessible locations can be treated more easily with nanotheranostic devices since they can penetrate biological barriers like the blood-brain barrier 19. In general, nanotheranostics that include herbal bioactives show promise as a method to treat a variety of disorders. To fully harness the medicinal power of herbal medicine in individualised healthcare, more research and development in this area is essential. The detailed description of various imaging techniques used for herbal nanotheranostics are shown in Table 2.

### 5.1. Theranostics nano vesicular of herbal compound for cancer targeting

#### 5.1.1. Theranostic polymeric nanovesicular of herbal compound

Nanoscale biocompatible polymer vesicles, or theranostic polymeric nanovesicles, are manufactured with great care to encapsulate medicinal herbal ingredients 98. Nanovesicles like this have a lot of properties that could be useful in personalised treatment. Researchers pick these herbal components for their anti-inflammatory, antioxidant, and anticancer benefits, among other known medicinal qualities 99. The chemicals are encapsulated within nanovesicles, which improves their solubility, stability, and controlled release. This enhances their medicinal efficacy and minimizes negative effects 100.

**Figure 7:**
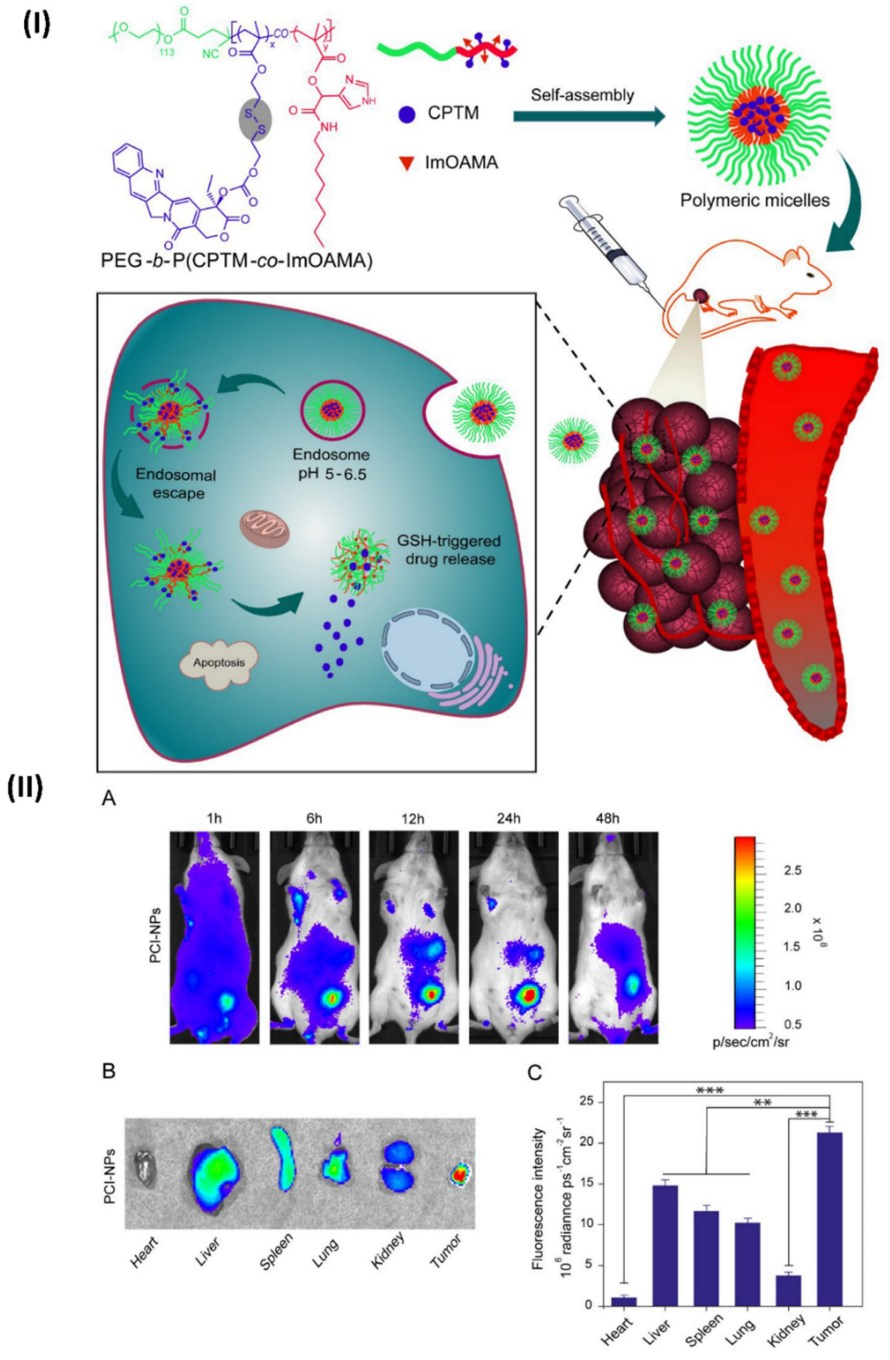
(I) Enhanced endosomal escape capability and prompted parent CPT drug release in the cytoplasm are depicted schematically in the pH- and reduction-responsive prodrug micelles; (II) (A) Examining the *in vivo* biodistribution of DiR iodide-loaded PCI-NPs using intravenous contrast imaging in BALB/c mice carrying the H22 tumour following intravenous injection. (B) After 24 hours of intravenous injection of PCI-NPs, fluorescent imaging of the tumour and key organs was performed on killed mice. (C) Assessment of tumour and primary organs following intravenous PCI-NP injection using semi-quantitative methods. Reproduced with the permission ref. 101 Scheme 2, and Fig. 5 (ACS Publications).

In a study, Ibrahim et al, developed polymeric micelles. The endosomes are internal organelles that trap PEG-bP(CPTM-co-ImOAMA) micelles once they have been internalised by tumour cells by endocytosis. The protonation of the imidazole groups of PImOAMA segments can be triggered by the endosomal pH. This could help the endosomes escape via the proton sponge effect and better contacts between the endosomal membranes, hydrophobic octyl groups, and protonated imidazole groups. Additionally, cancer cells' elevated glutathione levels in the cytoplasm can activate the release of active camptothecin (CPT) by rupturing the disulphide linkers in PCPTM. An important factor in the dramatically enhanced cancer cell killing efficacy was the ability of PEG-b-P(CPTM-co-ImOAMA) micelles to be efficiently internalised into cells and subsequently escaped by endosomal pathways, as demonstrated by the *in vitro* results. The PEG-bP(CPTM-co-ImOAMA) micelles effectively inhibited tumour growth without causing any noticeable side effects, according to *in vivo* investigations. With their high endosomal escape capabilities, the reduction-responsive polymeric prodrug micelles could be a game-changer in the drug delivery platform, leading to improved antitumor efficacy. In addition, mice were used to assess the *in vivo* biodistribution of PC-NPs and PCI-NPs, with a subcutaneously formed H22 tumour model (~100 mm3). In a nutshell, polymeric micelles were loaded with DiR probes, and the distribution of nanoparticles *in vivo* was tracked using *in vivo* visual imaging (IVI) using the emission of near-infrared fluorescent signals stimulated by 680 nm light. Following the intravenous injection of DiR iodide-loaded nanoparticles into a tumour site via the tail vein, researchers noticed a dramatic increase in the fluorescence intensity of DiR@PCI-NPs. This increase in intensity peaked around 24 hours after injection and persisted for up to 48 hours (Fig. 7IIA). Figs. 7IIB and 7IIC show the findings of an investigation into the *in vivo* fluorescence intensity profile that involved collecting tumours and major organs 48 hours after intravenous injection. The results showed that PCI-NPs efficiently accumulated and were retained in the tumours. Tumour fluorescence intensity was comparable in animals treated with DiR@PC-NPs. These findings provided compelling evidence that nanoparticles with the right ratio of hydrophilic to hydrophobic groups, owing to their stable size in systemic circulation and EPR effect, might accumulate in tumours 101.

In another study, Bleul et al., developed polymeric vesicles using micromixer technology and filled with magnetic nanoparticles and the anticancer medication camptothecin. Transmission electron microscopy verified the magnetic nanoparticles' successful integration. The size distribution of the hybrid polymersomes was found to be relatively limited in the dynamic light scattering tests. The cell survival of prostate cancer cells (PC-3) assessed after 72 hours was dramatically reduced by camptothecin polymersomes, whereas drug-free polymersomes did not exhibit any cytotoxic effects. The hybrid polymersomes were conjugated with a cancer targeting peptide (bombesin) and a fluorescent marker (Alexa Fluor 647). Flow cytometry and confocal imaging demonstrated targeted cell binding and internalisation. Relaxometry data showed that magnetic polymersomes can produce a noticeable amount of T2-weighted MRI contrast, which could pave the way for direct tracking of the polymersomes' biodistribution. A further step from fundamental research to personalised medicine is micromixer technology, which allows for the easy, quick, and efficient manufacturing of hybrid polymersomes as theranostic drug delivery devices 102.

#### 5.1.2. Theranostic hybrid nano vesicular of herbal compound for colon targeting

Theranostic hybrid nanovesicles are state-of-the-art nanostructures that can diagnose and treat medical conditions at the same time by utilising the medicinal properties of plant substances 103. A hybrid technique is used to construct these nanovesicles, optimising their properties by integrating a variety of components such as lipids, polymers, or nanoparticles 104, 105. The medicinal benefits of herbs can be fully utilised when their constituents are encapsulated within these nanovesicles, which improves their stability, bioavailability, and targeted administration 104. The diagnostic and therapeutic efficacy of these nanovesicles can be further improved by engineering them to react to particular disease biomarkers or stimuli. Their adaptability makes them a strong contender in the field of personalised medicine, which aims to address diseases and patients by creating unique treatments 106.

**Figure 8:**
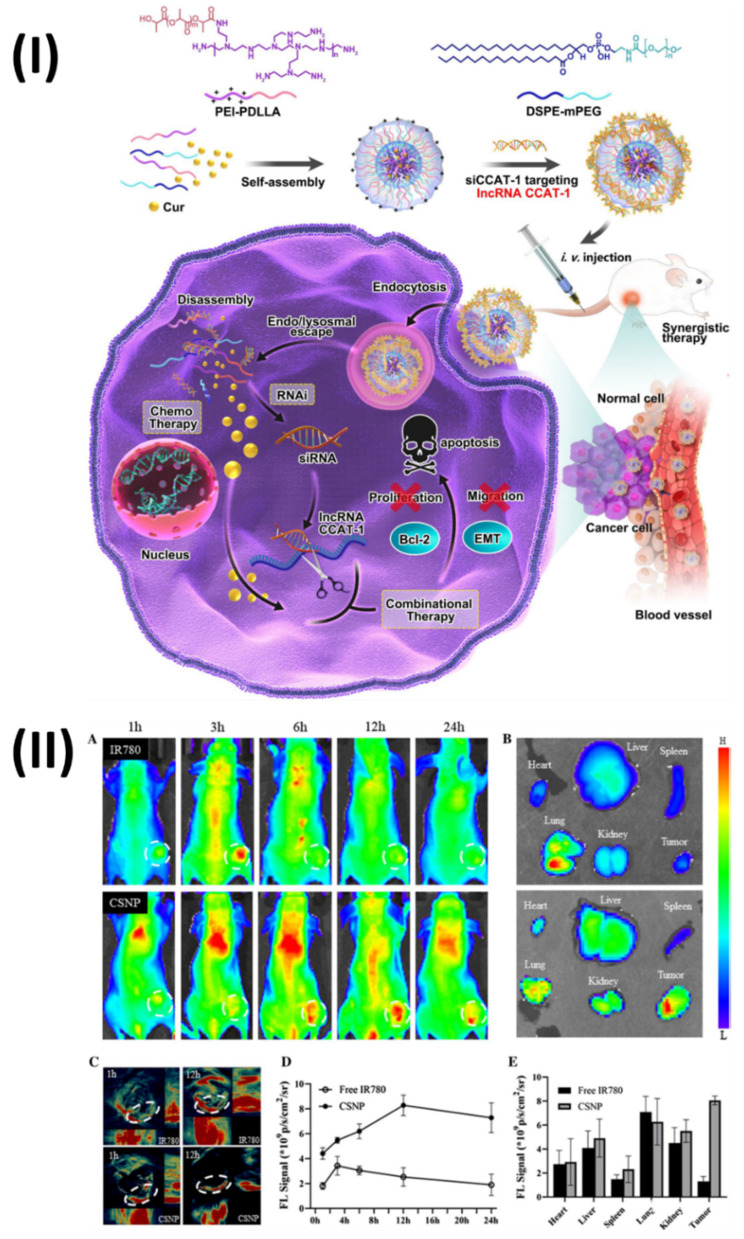
(I) A model showing the construction of a self-assembled micellar system for lncRNA CCAT1 silencing and Cur co-delivery using PEI-PDLLA and DSPE-mPEG. The regulation of multiplex CCAT1-related downstream genes allowed for the combinational therapeutic effects. (II) Fluorescence and photoacoustic imaging. Reproduced with the permission from ref. 107. Scheme 1, and Fig. 5 (Springer).

In a study, Jia et al, developed hybrid nanoparticles for colorectal cancer. Polymeric hybrid nanoparticles (CSNP) were created in this study by combining curcumin (Cur) with small interfering RNA targeting lncRNA CCAT1 (siCCAT1). The CSNPs were assembled using the self-assembling method with two amphiphilic copolymers, DSPE-mPEG and polyethyleneimine-poly (d, l-lactide) (PEI-PDLLA). Owing to PEI-PDLLA's multicolor fluorescence properties, the CSNP that was built could be used as a theranostic nanomedicine for simultaneous imaging and therapy in both laboratory and living organism settings. As a result, CSNP with coordination patterns effectively suppressed HT-29 cell growth and migration while inducing the highest apoptosis ratio. One interesting finding was the simultaneous regulation of important downstream genes and the effective suppression of lncRNA CCAT1. In addition, the HT-29 subcutaneous xenografts model demonstrated noticeable anti-tumor activity in response to CSNP, and the treatment was both safe and compatible with the patient's body. Fig. 8I showed the process for preparing the Cur and siCCAT1 co-delivery system of CSNP. Fig. 8IIA shows that following intravenous injection, the free IR780 fluorescence signal quickly accumulated at the tumour site within 3 hours and then faded away at 6 hours.

In contrast, the CSNP fluorescence signal strength at the tumour site increased gradually and reached its peak at 12 hours. Fig. 8IID shows that CSNP could constantly accumulate in the tumour site through the EPR, since the fluorescence signal of free IR780 could only endure for around 6 hours, in contrast to the long-lasting fluorescence signal of CSNP, which did not fade until 24 hours. At 24 hours post-injection, the mice were sacrificed and their vital organs were harvested for ex vivo fluorescence imaging. Fig. 8IIB shows that the CSNP group accumulated significant fluorescence near the tumour site, whereas the free IR780 group mainly enriched fluorescence in the lungs; the quantification analysis corroborated these findings (Fig. 8IIE). Furthermore, both fluorescence and photoacoustic imaging yielded the same results (Fig. 8IIC), indicating that CSNP tended to accrue enrichment at tumour regions and could be utilised as a self-monitoring agent for both types of imaging 107. Moreover, Unal et al., investigated the in vivo performance of optimised polycationic cyclodextrin (CD) nanoparticles for oral delivery of Camptothecin (CPT) in a tumor-bearing mouse model at both the early and late stages of tumour development. The efficacy of CPT bound to these nanoparticles was compared to that of free CPT in terms of antitumoral and antimetastatic effects. Furthermore, using in vivo imaging equipment and comparing the fluorescent dye intensity, they also photographed and evaluated the gastrointestinal localization of a single oral injection of polycationic cyclodextrin (CD) nanoparticles loaded with fluorescent dye at the end of 24 hours. The findings demonstrated that compared to the oral CPT solution, CRC-bearing mice had a considerably higher survival percentage, fewer colorectal tumour masses, and fewer liver metastatic foci. Additionally, in vivo imaging investigations have verified that polycationic CD nanoparticles can transport the therapeutic load to the colon and concentrate mostly in tumour foci, suggesting a successful targeted approach to treatment. The results showed that polycationic CD nanoparticles loaded with CPT could be a good oral nanocarrier formulation for anticancer compounds with low oral bioavailability and poor stability 108.

Moreover, Wang et al., created a new oral drug delivery system (DDS) using the non-toxic polymers chitosan and gelatin to create active targeted nanoparticles. Using a glutaraldehyde cross-linker, the nanoparticles had wheat germ agglutinin (WGA) conjugated onto their surface during their fabrication process, which involved a sophisticated coacervation procedure. To be more precise, we combined 5-fluorouracil (5-FU), the initial line of defense against colon cancer, with epigallocatechin-3-gallate (EGCG), a compound that suppresses tumour growth through anti-angiogenesis and apoptosis-inducing actions, into nanoparticles called WGA-EF-NP. Longer circulation time, improved cellular absorption, and prolonged drug release were all observed with the nanoparticles that contained both 5-FU and EGCG. Compared to medicines and nanoparticles without WGA ornamentation, WGA-EF-NP had stronger anti-tumor activity and pro-apoptotic efficacy because it was more bioavailable and had a longer circulation duration in vivo. This suggests that WGA-EF-NP could be an effective DDS for the treatment of colon cancer 109.

### 5.2. Theranostic quantum dots of herbal compound for bioimaging and therapy

Quantum dot (QD)-based strategies have found numerous uses throughout the years, including in nanomedicine, photoinduced therapy, drug transport, and fluorescence imaging. The biomedical field is showing interest in QDs because of their useful characteristics, which include biocompatibility, minimal toxicity, excellent electrical and chemical stability, and tunable fluorescence 110. A fortunate by product of material science is the discovery of quantum dots. Carbonaceous nanoparticles, measuring just 1-10 nm in diameter. Various polar functions, like tiny functional molecules, polymeric molecules, and so on, can be surface-decorated onto quantum dots due to their extremely polar nature. A state-of-the-art method in biomedical research, theranostic quantum dots (QDs) combine diagnostic and therapeutic capabilities on a single platform 111. Herbal chemicals have a wide range of pharmacological effects and relatively low toxicity profiles, which has piqued the interest of the theranostic community in recent years. Bioimaging and therapy might benefit greatly from the combination of herbal chemicals with QDs. Herbal chemicals, which are sourced from plants, can be used to diagnose and cure diseases because of their unique biochemical activity. These chemicals are promising for theranostic uses due to their antioxidant, anti-inflammatory, and anti-cancer characteristics. Researchers have developed multifunctional nanoparticles that can be used for targeted distribution, imaging, and therapy by encapsulating or conjugating these chemicals with QDs 112.

Sung et al., developed graphene quantum dots mediated theranostic drug and photolytic delivery via targeted biomimetic nanosponges. This research produced a red blood cell (RBC) membrane that mimics the structure of a sponge and is supported by carbon composites.

**Figure 9:**
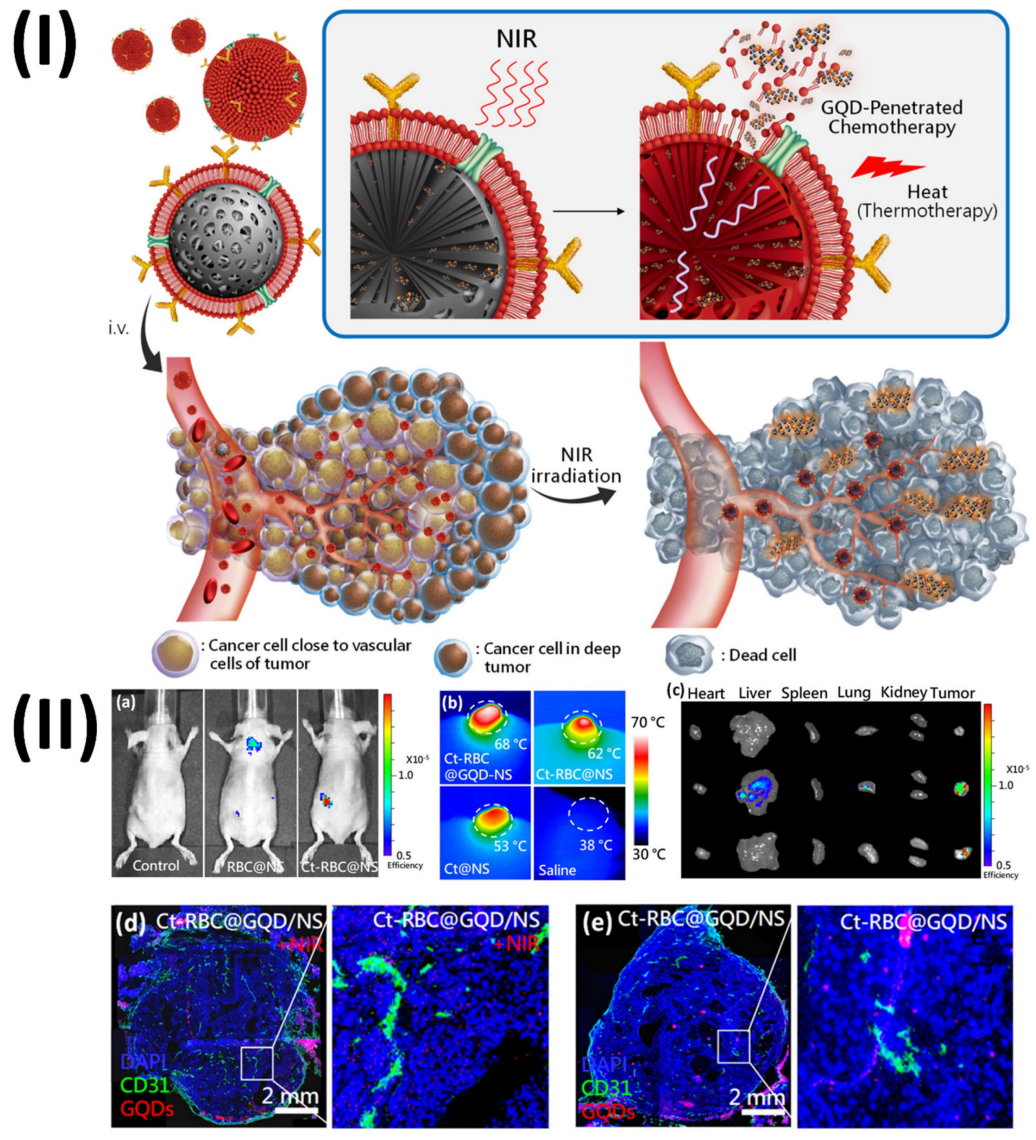
(I) Illustration of drug/graphene quantum dots (GQD) penetration and targeted RBC-membrane encased nanosponge-mediated tumour accumulation; (II) Investigation of NSs in live animals using BALB/c nude mice carrying the A549 tumour. Reproduced with the permission from ref. 113. Fig. 1, and Fig. 5 (ACS Publications).

The membrane serves as both a photolytic carrier that transfers heat and tumor-penetrative chemicals (GQDD and graphene quantum dots) when exposed to radiation. The RBC-membrane enveloped nanosponge (RBC@NS) with a targeted protein shows eight times the accumulation of the NS when it comes to tumour spheroids through high lateral bilayer fluidity. The process of delivering GQDs to tumour areas involves using near-infrared irradiation to activate a structure that is just one atom thick. This structure allows the medicine to penetrate deep into the tumour tissue. The theranostic GQDs efficiently delivered a combination of photolytic and chemotherapeutic actions to tumours, causing damage and inhibition of the tumour in as few as 21 days after a single irradiation. With its increased tumour targeting, NIR-induced drug penetration into tumours, and thermal ablation for photolytic therapy, this targeted RBC@GQD-D/NS demonstrates the potential for tumour suppression and other biomedical applications. Accumulation enhancement by targeting was demonstrated by the fluorescence intensity, which showed that Ct-RBC@NS had higher accumulation than RBC@NS (Fig. 9a). Afterwards, tumor-bearing mice that had been treated with particles were exposed to 1.5 W/cm2 of near-infrared light for 10 minutes to perform photothermal conversion. The tumour treated with Ct-RBC@GQD/NS can be heated to 68 °C, as shown in Fig. 9b. This temperature is suitable for thermal tumour ablation. Additionally, tumour temperatures rose to 62 °C and 53 °C after treatment with Ct-RBC@NS and Ct@NS, respectively. There was no discernible rise in temperature in the saline-treated mice following radiation. Higher accumulation and photothermal combined effects of GQD and NS may explain why the Ct-RBC@GQD/NS exhibits greater photothermal conversion. The next step was to use the IVIS to do spectral imaging of the primary organs and tumours. While both RBC@NS and Ct-RBC@NS fluoresced in the liver and lungs (Fig. 9c), the presence of particle metabolism in the clearance organs caused the accumulated particles at the tumour to show significant fluorescence. To assess the GQD penetration of particles exposed to 10 minutes of NIR irradiation (1.5 W cm^-2^) by immunohistochemistry (IHC), Fig. 9d displays fluorescence pictures of whole tumours. Red represents the GQDs, green represents blood vessels labelled with CD, and blue represents nuclei. It was clear that the GQDs had exceptional tumour penetration after NIR irradiation since their fluorescence signals reached almost every area of the tumour. The GQDs exhibited remarkable penetration behaviour, as seen in the magnified fluorescence image (Fig. 9d), which allowed them to reach depths of several hundred micrometres from the blood arteries. Tumours treated with Ct-RBC@GQD/NS displayed photothermal effects, such as cracks and voids, when exposed to near-infrared radiation. Alternatively, Ct-RBC@GQD/NS showed tumour aggregation in the absence of NIR irradiation (Fig. 9e) 113.

### 5.3. Theranostic nano vesicular of herbal compound for lysosomal dysfunction

Lysosomal dysfunction is a hallmark of many diseases, including neurodegenerative disorders and lysosomal storage disorders (LSDs), and theranostic micro vesicles filled with herbal chemicals offer a potential new approach to treating this problem. Natural substances with medicinal properties for lysosomal function are enclosed in these nano vesicles, which are usually made of biocompatible materials like lipids or polymers 114. Some herbal components, such as quercetin, resveratrol, or curcumin, have the ability to stabilise lysosomes by reducing lysosomal membrane permeability, increasing lysosomal enzyme activity, and modulating lysosomal pH 115. Several advantages are revealed by enclosing these substances in nano vesicles. First, precisely focused drug delivery is possible with the help of nano vesicles, which can be engineered to target certain cells or tissues impacted by lysosomal dysfunction. Second, lysosome-encapsulated herbal substances have a longer therapeutic influence because they are protected from degradation, which increases their bioavailability 116. The third point is that the therapeutic effectiveness against lysosomal dysfunction can be enhanced by combining different herbal substances in a nano vesicular formulation. This could potentially stop the progression of the disease. It is also possible to build nano vesicles to include diagnostic agents, which would allow for the imaging of lysosomal structures and functions concomitant with therapeutic intervention 117. Finally, theranostic treatments for lysosomal dysfunction have an improved safety profile due to regulated release from nano vesicles, which minimises off-target effects and systemic toxicity. In a nutshell, theranostic nano vesicles containing herbal compounds show enormous potential in treating lysosomal dysfunction; these vesicles provide targeted therapy, increased bioavailability, therapeutic synergy, and diagnostic capabilities; and they may even improve patient outcomes by advancing the treatment of related disorders 118.

Moreover, Li et al. demonstrated an activatable molecular assembly based on oxazine (PTO-Biotin NPs) that can induce ferroptosis by activating the lysosomal dysfunction-mediated fenton pathway. This assembly demonstrates exceptional spatiotemporal resolution when triggered by near-infrared (NIR) light. The heightened photothermal activity in the acidic microenvironment allows PTO-Biotin NPs to selectively target lysosome accumulation inside tumour cells. Activation of PTO-Biotin NPs by near-infrared light led to lysosomal malfunction, cytosolic acidification, and defective autophagy. Crucially, the anticancer efficacy was improved, and systemic adverse effects were mitigated through photoactivation-mediated lysosomal dysfunction via PTO-Biotin NPs, which remarkably increased cellular Fenton reactions and induced ferroptosis.

**Figure 10:**
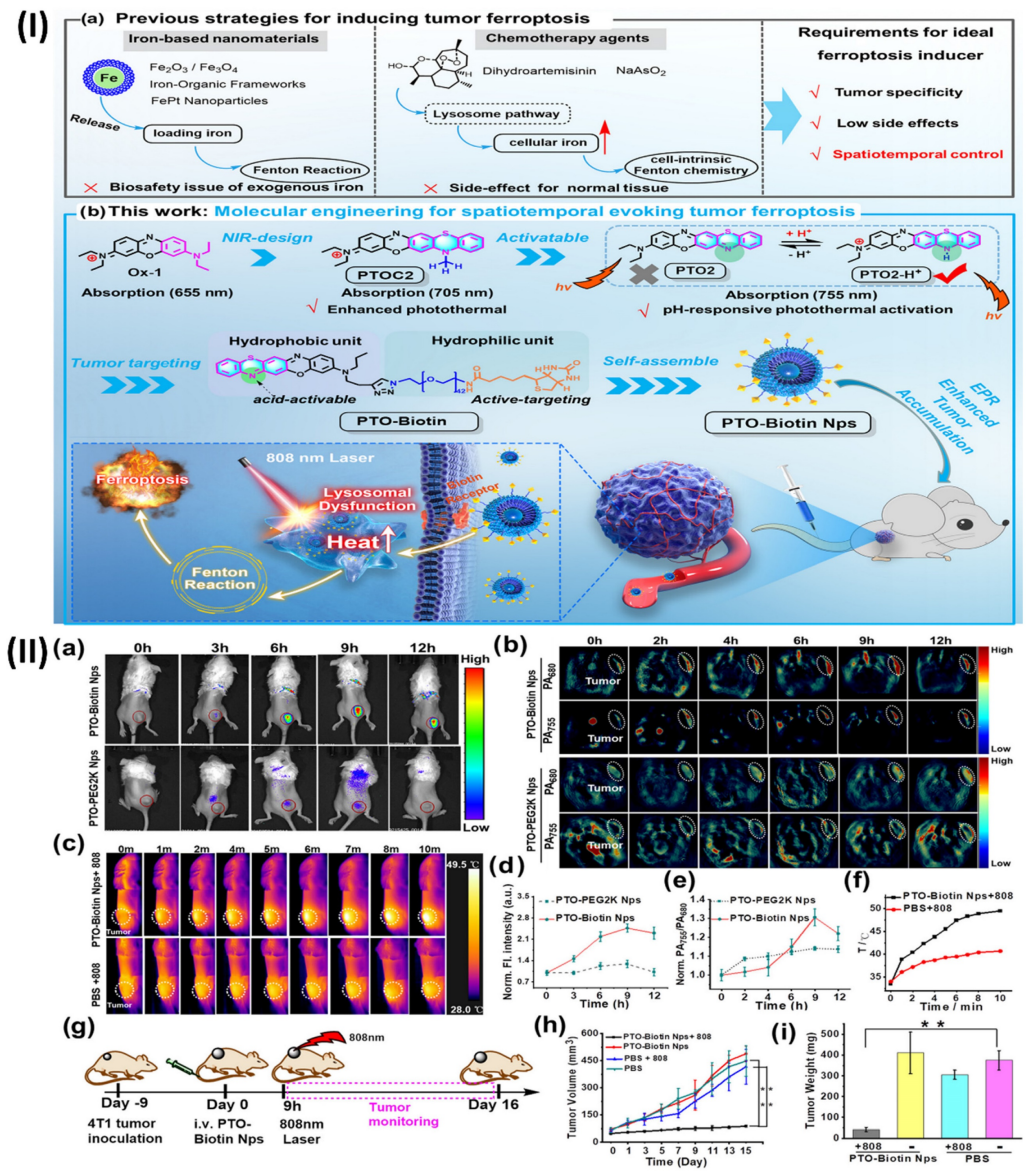
(I) (a) Methods Suggested for Causing Ferroptosis. (b) Current Approach to Spatiotemporal Inducing Tumour Ferroptosis via the Molecular Engineering of pH-Responsive Photothermal Oxazine Assemblies (PTO-Biotin NPs) (II) (a, b) Fluorescent or PA imaging was used to scan the 4T1 xenograft tumor-bearing Balb/c mice after they were intravenously injected with 300 μM or 100 μL of PTO-Biotin NPs or PTO-PEG2K NPs, respectively. (c) Intravenous injection of PTO-Biotin NPs (300 μM, 100 μL) or PBS, followed by 10 minutes of 808 nm laser irradiation, was used to acquire infrared thermal pictures of 4T1 tumor-bearing mice. (d, e) Panels (a, b) show the normalised fluorescence intensity and photoacoustic ratios (PA755/PA680), respectively. (f) The temperature change of panel (c). (g) Visual representation of the therapeutic trial. (h) Morphological patterns of many types of tumours (i) At 16 days after intravenous injection, tumour weight profiles were recorded for each group. Reproduced with the permission from ref 119. Scheme 1, and Fig. 6 (ACS Publications).

The outcomes of the research revealed that pH-responsive photothermal oxazine assemblies may be engineered through molecular engineering to modulate the intrinsic ferroptosis process in space and time. This opens up new possibilities for the creation of anticancer drugs that do not contain metal Fenton inducers. Prior to this, NIR fluorescence and PA imaging were used to investigate the tumour targeting capabilities of PTO-Biotin NPs. Fig. 10IIa shows that the fluorescence signal of the tumour locations rose to a maximum approximately 9 hours after PTO-Biotin NPs injection, and subsequently steadily decreased; this allowed for the ideal timing of NIR photoactivation in the tumour area to be determined. Alternatively, PTO-PEG2K NPs, the control chemical, displayed no change in tumour fluorescence signal intensity and disappeared after 12 hours. This suggests that PTO-Biotin NPs is more effective at targeting tumours than PTO-PEG2K Nps, which is not biotinylated. Because PA imaging penetrates deeper than fluorescence imaging, it also confirmed the enrichment effect of deep tumours (Fig. 10IIb, 10IIe) 119.

### 5.4. Theranostic nano vesicular of herbal compound for ulcerative colitis

**Figure 11:**
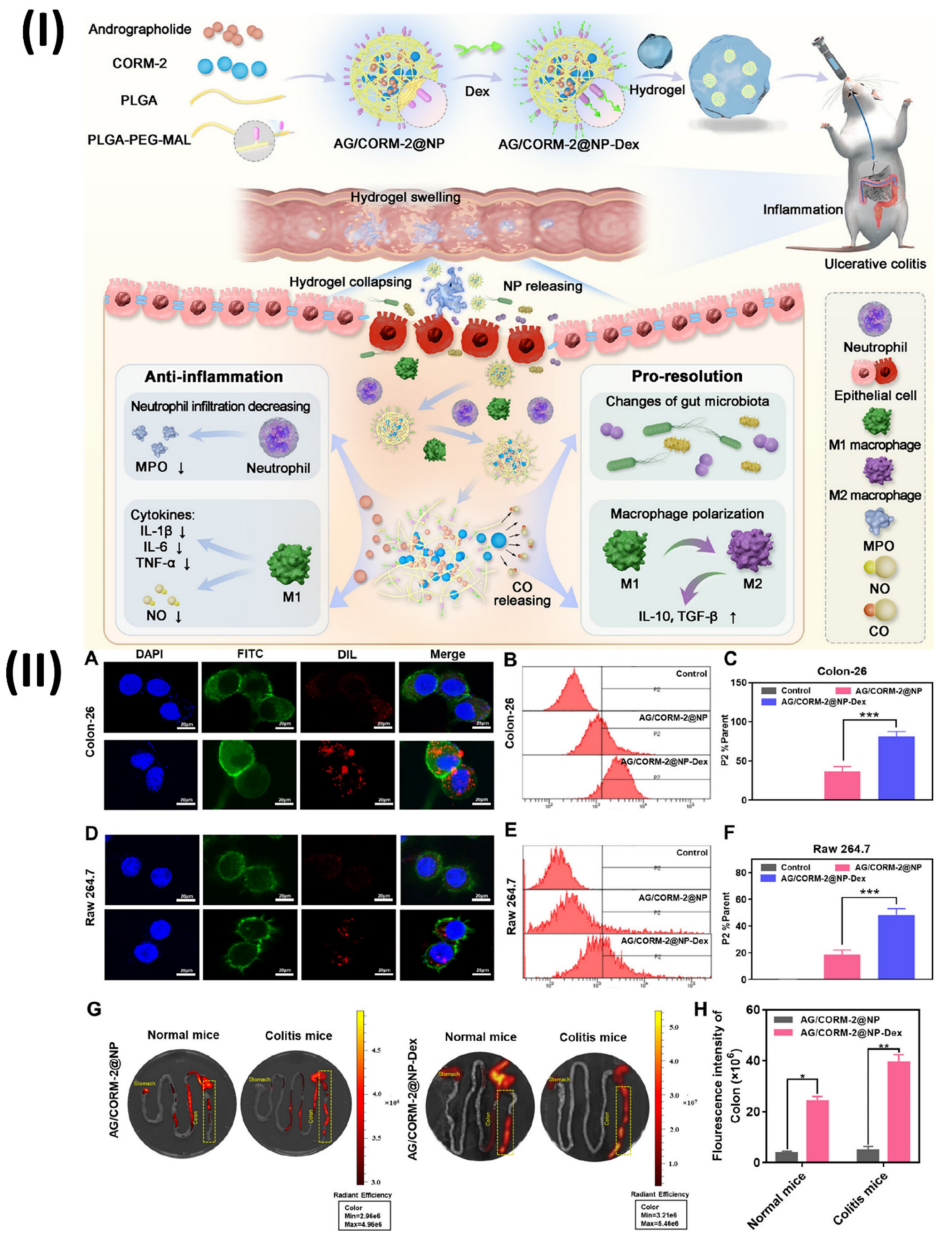
(I) A schematic depicting the oral nanotherapeutics of AG and the CO donor that work together to treat UC in a way that is both anti-inflammatory and productive in its resolution; **(II)** Targeting ability of AG/CORM-2@NP-Dex both *in vitro* and *in vivo*. Reproduced with the permission from ref. 125. Fig. 1, and Fig. 3. (ACS Publications).

An intriguing strategy for the management of ulcerative colitis, a chronic inflammatory bowel illness marked by inflammation of the mucosa and ulceration, is the use of theranostic nano vesicles that contain herbal components. While imaging and evaluating inflammatory bowel disease (IBD), imaging techniques can provide promising features 120. Modern advancements in multifunctional nanoparticles employ an interdisciplinary approach to guide IBD diagnosis. Early IBD diagnosis and disease severity monitoring are both made possible by the integration of nanotechnology with imaging technologies 121. This approach has potential applications down to the molecular level. The introduction of nanoparticles a class of manufactured small molecules with a wide range of biologically relevant characteristics has been a game-changer in the fields of disease detection, therapeutics, and theranostics 122. The nanomaterials show promise at extremely low concentrations and have less side effects than traditional medications due to their nanometer-scale dimensions, targeted delivery to inflammatory tissues, and controlled release 123. Herbal compound-loaded theranostic nano vesicles show great potential for the treatment of ulcerative colitis due to their diagnostic capabilities, sustained release, therapeutic synergy, improved bioavailability, and targeted therapy. Innovative treatments that enhance illness outcomes and quality of life for ulcerative colitis patients may be developed as a result of additional research and development in this field 124. A dextran-functionalized PLGA nanocarrier was created by Zhou et al. to efficiently deliver Andrographolide (AG) and carbon monoxide donor (CORM-2). This nanocarrier has several desirable properties, including biocompatibility, slow drug release, efficient targeting, and biodegradability; it was used in a synergistic anti-inflammatory/pro-resolving treatment of UC (AG/CORM-2@NP-Dex). A spherical shape and a nano-scaled diameter of approximately 200 nm characterised the resultant nanocarrier. Colon-26 and Raw 264.7 cells efficiently internalised the dextran-coated AG/CORM-2@NP-Dex in vitro, and the resultant AG/CORM-2@NP-Dex was able to be gavage to the inflammatory colon with chitosan/alginate hydrogel protection. The anti-inflammatory effects of AG/CORM-2@NP-Dex were achieved by reducing the overproduction of nitric oxide (NO), which is a pro-inflammatory mediator, and by down-regulating the expression of IL-1β, IL-6, and TNF-α. On the other hand, the pro-resolving function was demonstrated by the acceleration of M1 to M2 macrophage conversion and the upregulation of resolution-related genes, such as IL-10, TGF-β, and HO-1. Administering AG/CORM-2@NP-Dex in a chitosan/alginate hydrogel orally alleviated UC in the colitis model, owing to its synergistic anti-inflammatory and pro-resolving actions. This bifunctional nanocarrier is anticipated to accomplish long-term inflammatory remission without significant systemic damage, and it provides a new therapeutic approach for UC. The concentration of active compounds around the targeted tissue is improved by targeting delivery, which reduces injury to other essential organs and normal tissues. One example of a polymer is Dextran, or Dex. Around 95% of its composition consists of α-D- (1-6) connections. Dex can actively connect to cells expressing scavenger receptors and has an affinity for inflammatory disease areas. In light of these results, Dex may be utilised as a targeting ligand for the delivery of nanoparticles to the inflammatory colon. To find out if the targeting ability of AG/CORM-2@NP-Dex is enhanced by Dex modification, the cellular absorption profiles of AG/CORM-2@NP-Dex by Colon-26 and Raw 264 were examined. Using confocal microscopy, seven cells were examined qualitatively. After 6 hours of incubation with DiL-labelled AG/CORM-2@NP-Dex, confocal microscopy revealed that Colon-26 and Raw 264 successfully internalised the dye, as evidenced by a strong intracellular red fluorescence signal in seven cells (Fig. 11A, D). On the other hand, extremely low internalisation efficiency was shown by the absence of fluorescent signals from cells in the case of AG/CORM-2@NP without Dex alteration. Consistent with the confocal image results, flow cytometry assessed cell DiL fluorescence, suggesting that AG/CORM-2@NPDex could target Colon-26 and Raw 264.7 cells and had a stronger internalisation than AG/CORM-2@NP (Figs. 11B, 11C, 11E, 11F). The study utilised Colon-26 cells, a type of cancer cell produced from the colon epithelium in mice, to simulate the epithelial environment of colitis. Raw 264.7, a type of macrophage also derived from mice, served as the main effector cell in this inflammatory setting. Simultaneously, scavenger receptors are intrinsic immune recognition receptors that bind to various ligands, aid in the removal of altered self or non-self-targets, are abundant in tissue macrophages, and are vital in regulating inflammatory signalling. Curiously, the cellular absorption effect was enhanced in Raw 264.7 macrophages upon LPS stimulation. By modifying Dex, the absorption efficiency is enhanced, enabling the efficient delivery of AG/CORM-2@NP-Dex as a medication. After that, they looked at how AG/CORM-2@NP-Dex improved targeting in the inflammatory colon. A lipophilic cyanine dye that fluoresces near-infrared is known as DiR. Labeling cytoplasmic membranes and conducting near-infrared in vivo imaging are two applications of the dye. They tracked the nanoparticle's biodistribution using DiR. Mice with and without colitis were given a chitosan/alginate hydrogel containing DiR-labeled AG/CORM-2@NP and AG/CORM-2@NP-Dex. Fluorescence imaging using the IVIS imaging equipment was performed on the gastrointestinal tract and other important organs after 10 hours. In both healthy and colitis-stricken mice, AG/CORM-2@NP-Dex showed a stronger colon fluorescence signal than AG/CORM-2@NP, demonstrating that it could target the colon. Additionally, compared to normal mice, colitis mice show a more robust fluorescence intensity of the colon, indicating that the inflamed region was the preferential localization of AG/CORM-2@NP-Dex (Figs. 11G, 11H) 125.

In another study, Gao et al., identify the mechanisms underlying the development of turmeric loaded nanovesicles (TNVs) for the treatment of colitis. The TNVs were separated and made pure by using differential centrifugation. The IVIS imaging system was used to assess the targeted ability in a mouse model that was induced with dextran sulphate sodium (DSS). Researchers looked at the anti-inflammatory effects in a mouse model of acute and chronic colitic disease caused by lipopolysaccharide (LPS)-induced macrophages. Also, the state of macrophage polarisation after TNVs therapy was examined using flow cytometry, and the impact of TNVs on the intestinal microbiota was explored using 16S rRNA microbiome sequence. The results showed that TNVs were spheroids of nanometer size and were successfully separated. When administered orally, TNVs were able to aggregate at areas of inflammation in the colon and showed better anti-inflammatory action in both laboratory and animal studies. According to the 16S rRNA sequencing, TNVs play a crucial function in controlling the microbiota in the gut. Additionally, TNVs may be able to heal the damaged intestinal epithelial barrier and facilitate the change of M1 phenotype to M2 macrophages, both of which are necessary for the anti-colitis efficacy. In conclusion, by repairing the damaged intestinal barrier, controlling the gut microbiota, and changing the macrophage phenotype, TNVs administered orally demonstrated outstanding anti-inflammatory activity. The potential use of nanovesicles resembling natural exosomes in the therapy of UC is illuminated by this work 126.

### 5.5. Theranostic nano vesicular of herbal compound for kidney injury

Novel methods of renal disease theranostics have been developed using nanoparticles. The ability to regulate nanoparticles' size, shape, charge, and ligands makes them ideal for targeting the kidney. The use of nanoparticles in the fabrication of implantable artificial kidneys is also highly encouraging 127. Potentially harmful effects on cells and organs may result from exposure to various types of nanoparticles. Nanoporous membranes exhibit a size-dependent solute rejection as a result of their filtration properties. Potential implanted renal replacement solutions may be developed owing to these revolutionary advances in membrane technology 128. There are several ways to manage kidney injury using theranostic nano vesicles filled with herbal substances. These include focused therapy, improved bioavailability, sustained release, therapeutic synergy, diagnostic capabilities, and minimised adverse effects. Improvements in treatment results and quality of life for individuals with renal injuries could be achieved via ongoing research and development in this field 129.

Li et al., developed Gambogic acid (GA)-based nanoparticles (GA-NPs) with preferential renal uptake for AKI treatment. The improved accumulation of hydrophobic GA into approximately 4.5 nm nanoparticles was demonstrated in AKI models using PET scans, which were obtained by PEGylating with NH2-PEG5000-NOTA. Significantly, the nephroprotective properties and biosafety of GA-NPs have been validated by the in vivo tests of the two AKI models and in vitro cell assays. Hence, GA-NPs show promise as a therapeutic candidate for AKI therapy, according to this work. Based on the promising results of protecting cells with GA-NPs in vitro, it is expected that these particles can be put into damaged kidney tissue and protect it effectively. With PET imaging, the distribution and metabolism of probes in living organisms may be quantitatively visualised.

**Figure 12:**
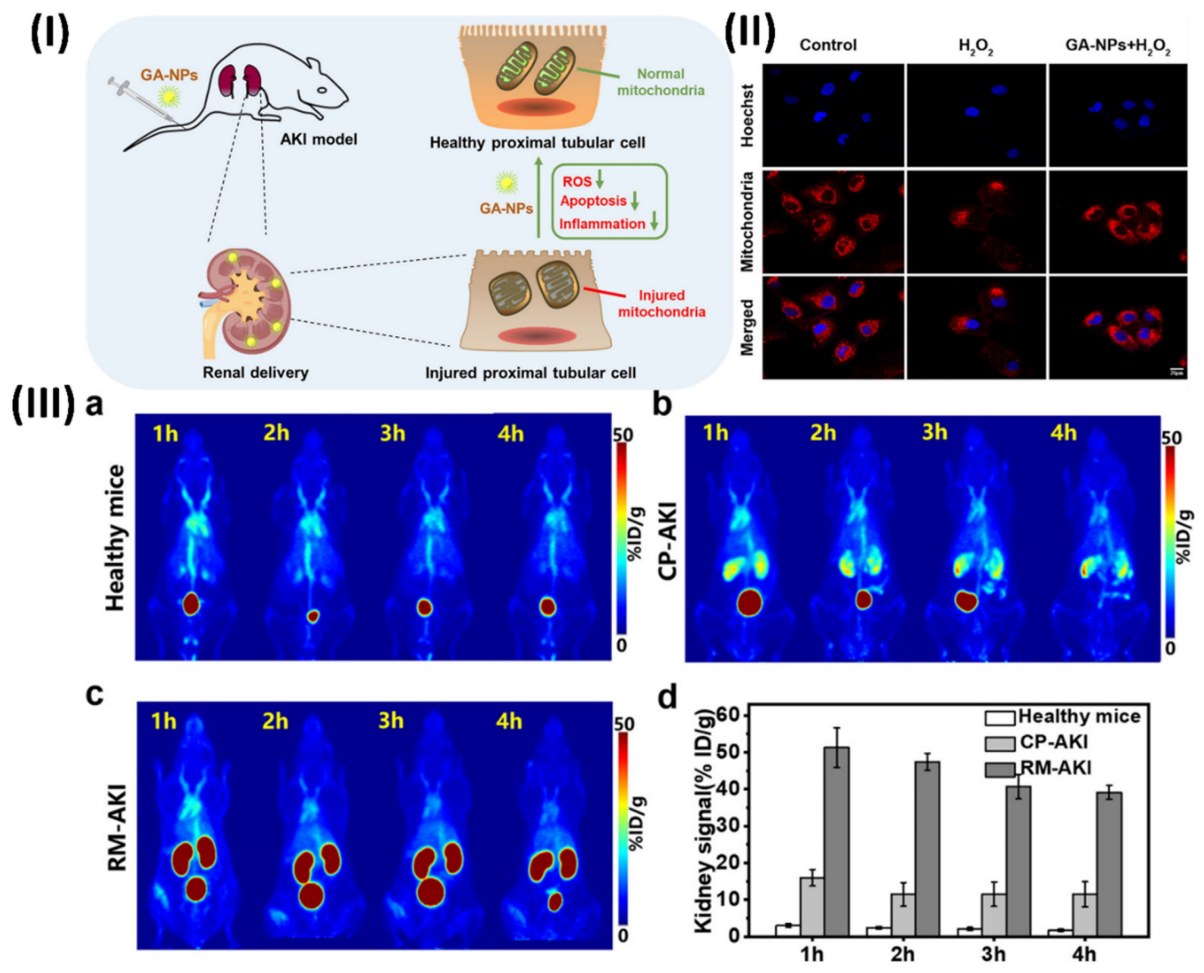
** (I)** Visual representation of GA-NPs that target the kidneys preferentially for acute renal injury treatment; **(II)** Incubation of HK-2 cells with GA-NPs (10 μg/mL) and H2O2 (350 μM) was used to stain the mitochondria depicted; **(III)** (a-c) Imaging studies using in vivo PET following administration of Al18F-GA-NPs in control, CP-AKI, and RM-AKI models of acute kidney injury; (d) Comparison of ROIs in two models of acute renal injury and in healthy mice. Reproduced with the permission from ref. 130. Fig. 1, Fig. 3, and Fig. 4 (ACS Publications).

Consequently, Al18F-labeled GANPs were evaluated by PET imaging on both healthy mice and two AKI-model animals, namely, cisplatin- and rhabdomyolysis-induced AKI. Water deprivation and intramuscular injection of glycerol were used to produce the RM-AKI model, while intraperitoneal injection of cisplatin was used to establish the CP-AKI model. Typically, nanoparticles that are specifically designed to target the kidneys make their way into the system via the bloodstream, traverse the glomerular filtration barrier, and ultimately be eliminated through urine. In healthy mice, the rapid elimination of Al18F-GANPs from the kidneys after injection was attributed to the small size of the particles, which was below the kidney filtration threshold (approximately 6 nm). In contrast, two AKI-model mice displayed increased retention of Al18F-GA-NPs in the kidneys at different time points, which can be explained by the blocking effect of injured kidneys (Figs. 12IIIa-c) 130.

Subsequently, Yuan et al., created a metal-phenolic nanozyme called RosA-Mn NPs that has antioxidant cascade capabilities. This nanozyme possesses a wide range of reactive oxygen species (ROS) and free radical scavenging capabilities in addition to its multienzymatic cascade capacity. As predicted, RosA-Mn NPs effectively scavenge ROS in vitro, which promotes mitochondrial function recovery while reducing endoplasmic reticulum stress and subsequent inflammation. Also, artificial enzymes have not been able to quickly accumulate in renal tissues, although RosAMn NPs solve that problem. A potential strategy to reduce tubular damage, enhance tubular functions, and halt the development of AKI could be to use the renal tubular targeting of RosA-Mn NPs. It is worth mentioning that in a mouse model of cisplatin-induced AKI, RosA-Mn NPs also shield the kidneys against oxidative stress and inflammation. RNA sequencing research showed that RosA-Mn NPs therapy regulated pathways associated to autophagy, apoptosis, and antioxidation. Overall, RosA-Mn NPs have promising therapeutic potential for AKI and other disorders associated with ROS due to their biocompatibility and multienzymatic activities 131.

### 5.6. Theranostic nano vesicular of herbal compound for liver fibrosis

There are several different types of liver injuries that can develop reversible hepatic fibrosis, which in turn can cause more serious problems like cirrhosis, liver failure, or even cancer of the liver. A lack of efficacy in treatment and unwanted side effects are two of the many problems with conventional pharmaceuticals 132. There has been a promising use of nanotechnology in the delivery of drugs for liver fibrosis. Enhanced internalisation and penetration made possible by nanomedicine pave the way for theranostics, combination therapy, and targeted drug delivery 133. A major health concern on a global scale is liver fibrosis, which is characterised by the increasing scarring of liver tissue. Chronic inflammation and injury to the liver can cause it to malfunction, which in turn can lead to cirrhosis or liver failure. One potential strategy for dealing with this complicated condition is the use of theranostic nano vesicles that contain herbal components 134. Utilisation of nano vesicles, which are vesicular structures on the nanometer scale and usually made of biocompatible components such as lipids or polymers, is central to this novel technique. The hepatic stellate cells are crucial in the advancement of liver fibrosis, and these nano vesicles encapsulate herbal substances that are known for their hepatoprotective, anti-inflammatory, and antioxidant characteristics, making them ideal for targeted delivery 135, 136. The nanovesicles targeted delivery feature maximises the therapeutic efficiency of the herbal components by precisely localising them within the fibrotic liver tissue, while minimising systemic exposure and any side effects. In addition to increasing bioavailability, encapsulation in nano vesicles shields herbal substances from degradation and makes it easier for liver cells to absorb them 137. With this improved delivery system, more therapeutic medicines can reach the damaged liver tissue, increasing their ability to slow the advancement of fibrosis. One great thing about theranostic nano vesicles is that they can release herbal components gradually over a long period of time 138. The anti-fibrotic and hepatoprotective benefits of individual herbal components can be enhanced by mixing them in a nano vesicular formulation, which allows for the utilisation of synergistic effects 106. Furthermore, these theranostic nano vesicles can be modified to include diagnostic agents or biomarkers, enabling diagnostic imaging-based non-invasive monitoring of fibrosis progression and therapy response. Clinicians can optimise patient care by making educated decisions about therapy changes because of these diagnostic capabilities 139.

**Figure 13:**
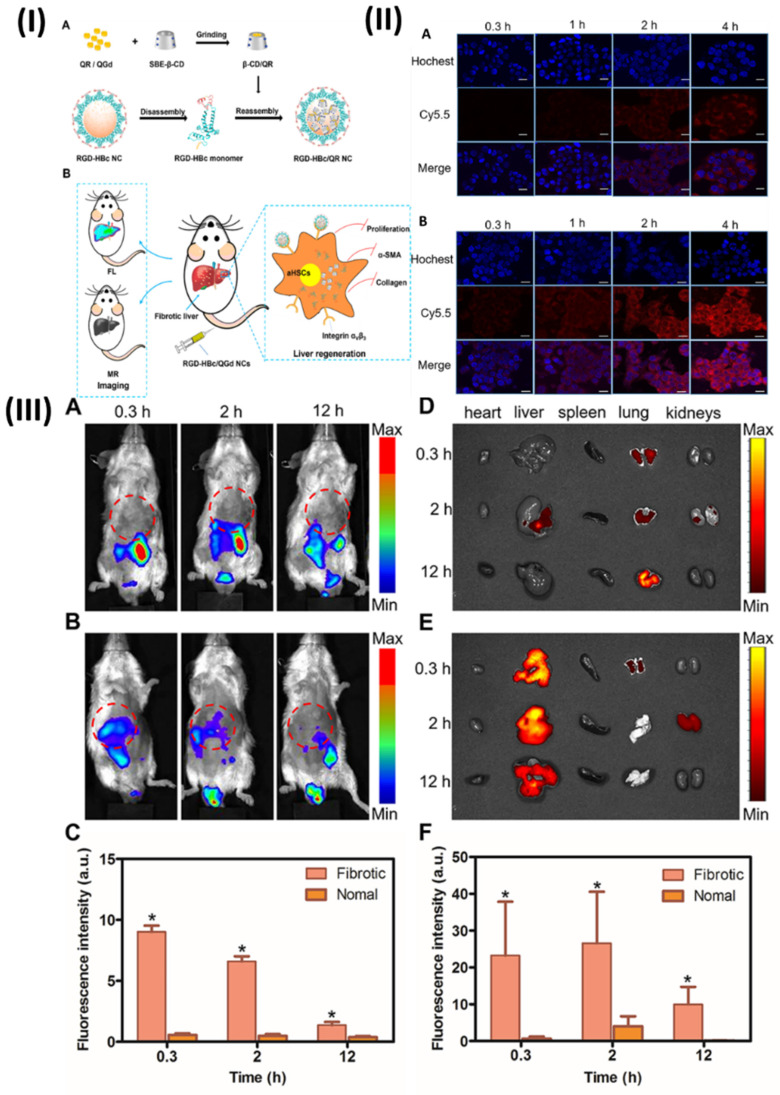
** (I)** (A) Self-assembled method for encapsulating QR/QGd into RGD-HBc NCs (B) Theranostic property of RGD-HBc/QGd NCs:* in vivo* FL and MR imaging of fibrotic liver (Left) and liver regeneration by aHSC proliferation (Right); **(II)** (A) aHSCs (B) treated with RGD-HBc NCs that had been labelled with Cy5.5 at the specified intervals; **(III)** Images of the tissues and in vivo mice imaging. Reproduced with the permission from ref. 140. Scheme 1, Fig. 3, and Fig. 5 (ACS Publication).

In a study, Zhang et al. encapsulated a natural medicine called quercetin (QR) into hepatitis B core (HBc) protein nanocages (NCs) for imaging and targeted treatment of hepatic fibrosis. In both laboratory and animal studies, it was found that nanoparticles (RGD-HBc/QR) containing a surface-displayed RGD targeting ligand effectively inhibit the proliferation and activation of hepatic stellate cells (HSCs), and they show a relatively high selectivity towards activated HSCs through their binding affinity with integrin αvβ3. The resultant nanoparticles (RGD-HBc/QGd) possess excellent potential as NIR fluorescent and magnetic resonance imaging contrast agents for hepatic fibrosis *in vivo* if they are enclosed in a quercetin-gadolinium complex and/or labelled with the NIR fluorescent probes (Cy5.5). Thus, the multifunctional integrin-targeted nanoparticles have the potential to offer an efficient antifibrotic theranostic approach by selectively delivering QR to the activated HSCs. Fig. 13IIA, 13IIB show that Cy5.5-labeled RGD-HBc NCs fluoresced red, while DAPI-stained cell nuclei fluoresced blue. In general, the merge area showed that RGD-HBc NCs could bind to aHSCs faster than HSCs cells, even when the uptake amount was enhanced throughout time. Liver fibrotic mice were injected intravenously with Cy5.5-labeled RGDHBc/QR NCs and their precise biodistribution was assessed at the designated time points. Mice exhibited significant liver fibrosis following 8 weeks of CCl_4_ therapy. The kidneys of healthy mice quickly removed RGD-HBc/QR from their blood, as seen in Fig. 13IIIA. 13IIIB, 13IIIC show that RGD-HBc/QR has a strong affinity for aHSCs and that, in contrast, it accumulates significantly and for a long period in the fibrotic liver. Furthermore, at the specified time points (0.3, 2, and 12 h) following intravenous injection, the fibrotic group had a greater fluorescent signal intensity in their ex vivo livers (Fig. 13IIIE) compared to the normal group (Fig. 13IIID). Measurement of the ex vivo livers' fluorescence intensities shown in Fig. IIIF. These data indicate that RGD-HBc/QR NCs can be successfully maintained in fibrotic livers by RGD-mediated active targeting 140. In another study, Wu et al., formed a ratiometric nanoprobe that can detect hepatic injury caused by the herbal remedy polygonum multiflorum (PM) in vivo. It works by responding specifically to the NO generated in the liver by PM, and when combined with MSOT imaging, they can pinpoint exactly where the injury is occurring. The liposomal nanoprobe is made up of a diketopyrrolopyrrole-based conjugated polymer (DPP-TT) serving as the internal reference and a responsive dye (IX-2NH2) that may selectively react to NO. Consequently, they may achieve noninvasive ratiometric optoacoustic detection of herbal medicine-induced liver disease using 3D data in a mouse model 141.

In another study, Luo et al., created silibinin albumin nanocrystals (SLB-HSA NCs), a nanoplatform for the treatment of liver fibrosis by targeting aHSCs. The SLB-HSA NCs that were synthesised demonstrated a consistent distribution of particle sizes, around 60 nm, with a PDI less than 0.15 and a loading efficiency of up to 49.4 percent. Evidence suggests that albumin-coated nanocrystals enhance aHSC cellular absorption via SPARC-mediated endocytosis. When tested in a pharmacokinetic trial alongside free SLB, SLB-HSA NCs considerably increased bioavailability. After being injected into the tail vein, SLB-HSA NCs accumulated heavily in the fibrotic liver and showed improved antifibrotic effects in animals with hepatic fibrosis. Thus, the results demonstrate the significant promise of SLB-HSA NCs for the specific management of liver fibrosis 142.

### 5.7. Theranostic nano vesicular of herbal compound for Rheumatoid Arthritis

Among the most common inflammatory-autoimmune disorders, rheumatoid arthritis (RA) is agnogenic and causes chronic joint abnormalities, cartilage loss, and persistent synovitis; it also increases mortality, causes a variety of disabilities, and places a heavy burden on society 143. By integrating diagnostic and therapeutic capabilities into a single platform, theranostic nano vesicles present a potential strategy for the treatment of RA. With fewer adverse effects than traditional treatments, these nano vesicles contain herbal ingredients with immunomodulatory and anti-inflammatory activities, making them a promising alternative for RA management 144. Herbal ingredients like quercetin, resveratrol, and curcumin have strong anti-inflammatory properties that can reduce RA symptoms by regulating immune responses and reducing inflammatory pathways. Treatment efficacy and systemic toxicity can be maximised by encapsulating these chemicals in nano vesicles, which improves their stability, bioavailability, and targeted administration to inflammatory joints 145. The incorporation of imaging probes or biomarkers into theranostic nano vesicles further enables real-time monitoring of illness development and therapy response. By serving two purposes, treatment can be tailored to each patient's unique needs, maximising positive results while reducing negative ones. Additionally, a natural and holistic approach to managing RA can be achieved through the use of herbal ingredients, which decrease the need for synthetic medicines 146. Safe, effective, and individually tailored treatments for rheumatoid arthritis may soon be within reach due to theranostic nano vesicles, which combine the synergistic effects of various herbal components 106.

**Figure 14:**
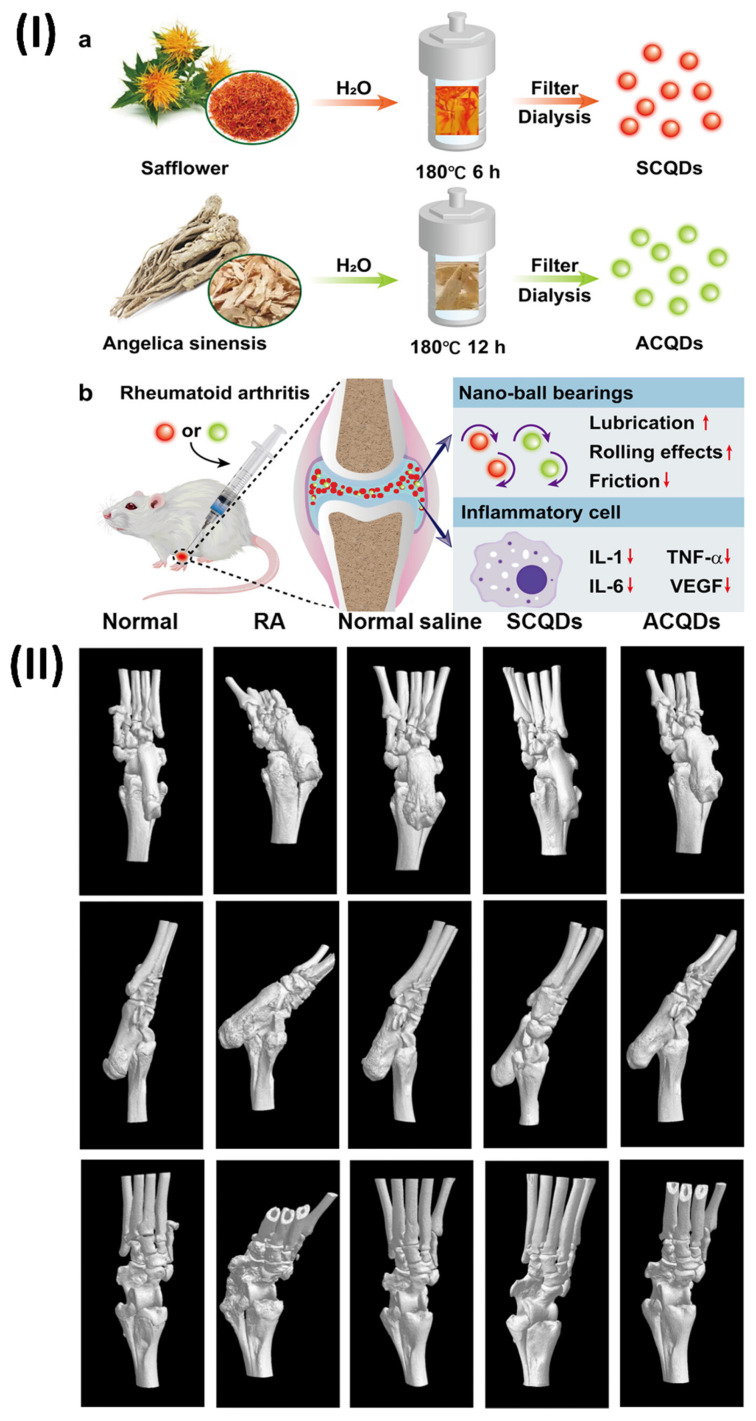
(I) Schematics showing (a) the production of SCQDs and ACQDs and (b) the in vitro and in vivo research studies; (II) Images of the right hind ankle joints, as reconstructed using micro-CT scans, taken on day 21 following various treatments in both healthy and diseased mice. Reproduced with the permission from ref. 147. Fig. 1, and Fig. 5 (ACS Publication).

In a study, Qiang et al., developed carbon quantum dots of herbal molecules for the treatment of rhematiod arthirits. Two new therapeutic nanoagents have been rationally developed using carbon quantum dots (CQDs) derived from herbal medicine, specifically safflower (Carthamus tinctorius L.) and Angelica sinensis. These nanoagents have ultrahigh lubrication and anti-inflammatory bioefficacy, which is ideal for the treatment of rheumatoid arthritis. Because of their spherical shape and high water solubility, the two nanoagents exhibit remarkable friction reduction in in vitro experiments. The results showed that the nanoagents effectively reduced swelling symptoms and impaired the production of associated inflammatory cytokines, such as IL-1, IL-6, and TNF-α, when tested on rat models of rheumatoid arthritis. New nanoagents for rheumatoid arthritis treatment could be developed by combining the lubricating and anti-inflammatory bioefficacy of CQDs generated from herbal medicine (Fig. 14) 147.

## 6. Pre-clinical importance of herbal theranostics in drug development

The theranostics nanomaterials are incredibly diverse, with a wide variety of clinical indications that call for specific administration routes, formulations for delivery, and technical details for experimental testing of the associated theranostics properties. As a result, there is no simple "one size fits all" solution for the preclinical evaluation of safety and efficacy in vivo 148. Some examples of such applications are multifunctional or multimodal imaging, image-guided surgery, photodynamic treatments, magnetic and environmental hyperthermia, and improved magnetic resonance imaging. Theranostics nanoparticles are classified as both medicinal items and medical devices, so it is exceedingly challenging to attempt to derive any universally applicable in vivo study design or findings on the substance 149. Therefore, it is necessary for the theranostic section to determine the PMoA of the nanomaterials and then couple them with an external medical device that provides multifunctionality and multimodality. The next step, after the national regulatory agency has identified and categorised it, is to follow the specific requirements for preclinical assessment based on in vivo evidence 150. The evaluation of pharmaceutical items is done as per the Pharmacopoeia recommendations, which include the evaluation of biodistribution, pharmacokinetics, pharmacodynamics, and efficacy, as well as maximal tolerable toxicity 151.

## 7. Regulatory and safety concerns of herbal theranostics

Medical products that are part of nanomedicine can be either biological (obtained from plants or animals) or non-biological (made from chemicals). The medicine must pass numerous quality tests concerning its safety, effectiveness, bioequivalence, and therapeutic equivalency before being released to the public. Before entering the market, certain pharmaceuticals or items must pass quality checks and financial controls set by individual countries. The principal regulatory agencies that provide guidelines for comparative quality, clinical, and non-clinical investigations are the European Medicines Agency (EMA) and the United States Food and Drug Administration (FDA) 152. To ensure that nanoformulations that can affect kinetic parameters and toxicity retain their particle properties, homogeneity and stability, biodistribution, and intracellular concentration, the EMA established guidelines for new drug developers to follow. Furthermore, a multidisciplinary method called Health Technology Assessment (HTA) is being established to assess the efficacy, safety, and cost-effectiveness of medicines to ensure that patients have access to them 153. There are two acts that the US-FDA uses to regulate nanomedicines: the Public Health Service Act (PHSA), which deals with therapeutic items generated from biological sources, and the Food, Drug, and Cosmetic Act (FDCA), which deals with pharmaceuticals and medical devices that are chemically produced. In their nanotechnology guidance documents, the US Food and Drug Administration (FDA) discussed several topics, including a risk-based framework for nanotechnology, manufacturing, quality controls, and particular environmental considerations for biological and drug products that contain nanomaterials 154. Current guidelines are solely for conventional herbal medicines, even though there are some in place to govern the requirements for pre-market approval, post-market inspection, product quality system management, etc., for herbal medicines in places like the EU, Africa, Korea, and the Philippines. There are no regulatory standards for traditional herbal remedies in India, for instance, due to their lengthy history of use. In light of the recent uptick in the use of herbal nanotheranostic, the benefits these have over traditional herbal formulations (along with the associated safety concerns), and the pressing need for regulatory guidelines and monitoring bodies to ensure the quality of herbal nanotheranostic, at every stage of their production, administration, and subsequent analysis 155.

## 8. Challenges in nanotheranostics comprising of herbal molecules

Nanotheranostics containing herbal compounds have great promise for healthcare-targeted treatment and diagnostics. However, there are several barriers that prevent their execution 156. One issue that nanotheranostic systems can solve is the poor solubility or rapid metabolism of herbal compounds, which can compromise their bioavailability. These systems can enhance absorption and provide protection. Incorporating targeting ligands into nanotheranostics is essential to ensuring the efficacy of these compounds by ensuring their selectivity towards target cells or tissues 157. Thorough preclinical investigations are necessary to address safety concerns related to immunogenicity and toxicity. Research on herbal drugs has several obstacles that must be overcome. The phytoconstituents found in plant extracts can range in molecular weight, solubility, medicinal potential, and chemical classes, and they are often mixed in a variety of ways. Some, like glycosides and polyphenolics, are polar and soluble in water; others, like monoterpenes, sesquiterpenes, diterpenes, tri-terpenoids, and so on, are non-polar and soluble in lipids; and yet others, like alkaloids and flavonoids, may be partially soluble in water or lipids and have a median polarity 158. All things considered, these differences make nanoformulation's design and characterization techniques more challenging. The use of bioassays to guide the fractionation of these extracts, however, can circumvent these difficulties. The synergistic effect might be kept because the improved fractions would only have phytoconstituents with the same chemical classes and/or solubility profiles. Biological barriers, such as the blood-brain barrier (BBB), limit the transport of large or hydrophilic drug molecules inside the brain 159. Environmental factors, like dense stroma, cause poor perfusion and drug distribution inside solids. Leaky microvasculature, on the other hand, limits the transport of drug molecules into the interstitial space. Thus, additional research into other advanced nanoformulation technologies, such as SLN, liposomes, polymer micelles, NLC, and phytosomes, is necessary to ensure that the medications reach their intended targets 160. Furthermore, it can be beneficial to improve efficacy and decrease toxicities and side effects by providing an active biological transport system for medication administration. Nanoformulation has many benefits, but it also comes with some concerns about toxicity 161. Serious concerns such as cardiac distress, anaphylactic shock, facial swelling and flushing, headache, etc. Because of this, before doing preclinical or clinical research, it is wise to conduct appropriate ex vivo or in vitro tests of possible risks, such as cytotoxicity, immunotoxicity, and genotoxicity, to rigorously assess the nanosafety 162. Further, to evaluate the potential short-term and long-term toxicity as well as biocompatibility issues linked to nano-sized drug delivery of herbal compounds, it is necessary to conduct specialised in vivo toxicology studies using suitable animal models 163. Innovations in nanomedicine formulations that can tap into the medicinal properties of herbal compounds for better health outcomes can only be achieved through the combined efforts of pharmacologists, chemists, material scientists, engineers, and physicians.

## 9. Conclusion and future perspective

Herbal medications, which are produced from plants, provide a more holistic approach to healthcare; yet, there are several limits to be aware of such as standardization, safety concern, efficacy, delayed action, regulatory challenges etc. Herbal treatments have been used for medicinal purposes worldwide since ancient times. Both medical professionals and patients agree that these medications are more effective than conventional ones, and they also have fewer adverse effects. The herbal drug market needs more openness, better regulation, and standardised testing procedures to fix these issues. Both healthcare practitioners and consumers might have more faith in herbal medications as a form of alternative or complementary medicine if quality control processes are put in place to guarantee that they are safe and effective. The risk of side effects and drug interactions is a major concern when it comes to herbal compounds that have direct contact with patients. Herbal remedies have bioactive components that can have pharmacological effects on the body, despite their common perception as being harmless and all-natural. A scientific approach is necessary to provide the components in a sustained manner, which will increase patient compliance with phytotherapeutics such as camptothecin, rutin, artemisinin, curcumin, silymarin, gambogic acid etc. and lessen the need for repeated administration. To accomplish this, one needs to develop NDDS tailored to the components found in herbs. Reduced dose frequency due to non-compliance is just one benefit of NDDSs; they also aid in increasing therapeutic value by lowering toxicity and increasing bioavailability. A state-of-the-art approach lies in nanotechnology. Nanoscale herbal drug delivery systems have the potential to make herbal medicines more effective while reducing their downsides. A scientific approach is necessary to provide the components in a sustained manner, which will increase patient compliance with phytotherapeutics and lessen the need for repeated administration. Making NDDSs tailored to plant components is the key to accomplishing this aim. Additionally, NDDSs such as SLNs, NLC, niosomes, liposomes, polymeric nanoparticles, etc. enhance a drug's therapeutic efficacy by lowering its toxicity, increasing its bioavailability, and reducing the frequency of dosing needed to overcome non-compliance. According to recent advancements in nanomedicine, the technology that can detect diseases and potentially combine treatment and diagnostics is becoming a reality. Finally, for medications that aren't highly water-soluble or bioavailable, pharmaceutical nanotechnology is a new area of study that's improving upon traditional methods in every way. Efficient medicinal potency with minimal side effects is achieved by systems based on nanotechnology, which also improve targeted and sustained delivery of the entrapped substance. Theranostic nanomedicines provide a thorough and accurate way to manage these illnesses by providing a controlled and targeted approach to diagnosis and treatment. For various reasons, some novel nanoparticles have not progressed past the preclinical stage, while others have not been able to go on from in vivo studies to clinical trials. Also, some have argued that finding new, effective treatments for diseases is difficult because existing animal models don't accurately reflect human diseases. The clinical translation of nanomedicine and theranostics nanomaterials containing herbal molecules could benefit from the development of animal models. The treatment, and prevention of various diseases with herbal molecules in the future will be greatly assisted by theranostic nanoparticles that can simultaneously do imaging and therapy.

## Funding

This work received funding from Indian Council of Medical Research (ICMR), New Delhi, Govt of India (Project No. 35/8/2020-Nano/BMS).

## Figures and Tables

**Table 1:** Illustration of several nanocarriers for herbal actives

Nanocarriers	Preparative Method	Herbal actives	Composition	Key Findings	References
Nanosuspension	High speed homogenization	Paclitaxel (PTX)	Poloxamer 188, Mannitol	The bioavailability of PTX were increased when formulated as nanosuspension also the dissolution study confirmed it sustain release activity when compared to PTX alone	34
Solid Lipid Nanoparticles (SLNs)	High speed homogenization	β-Carotene	Stearic acid, Sodium taurocholate, Lecithin	The results indicated that SLNs are the commercially viable option for drug delivery due to its robustness. Researcher also shown the effect of mixing speed and time on size and polydispersity of nanoparticles.	35
Chitosan nanocomposite	High speed homogenization	*Pinus roxburghii*, *Juniperus communis*, and Cupressus *Sempervirens* Essential oils	Chitosan, Sodium Tripolyphosphate, Tween-80	The antifungal, antiaflatoxigenic and antioxidant activity of synergistic formulation of PJC increased when encapsulated within chitosan nanocomposite	36
Solid lipid nanoparticles	High speed homogenization	Gac oil (Momordica Cocochinensis Spreng)	Naterol SE, Span 80, Tween 80	The impact of Gac oil on particle size and stability of SLNs were demonstrated under UV radiation and storage temperature. The samples shown a colour change at high storage temperature as compared to low storage temperature	37
Microcapsule	Complex coacervation	Rutin	Alginate, Chitosan	With the desired entrapment efficiency, floating alginate-chitosan microcapsules are appropriate for use in Chinese materia medica's pulsatile drug delivery method.	38
Microspheres	Coacervation-phase separation	Salicylic acid (SA)	Chitosan, Poly(vinyl alcohol) (PVA)	SA release from the microspheres was shown to rise when the CS/PVA ratio dropped, but declined with the increased degree of crosslinking. Additionally, drug release at pH 1.2 was significantly greater than at pH 6.8 and 7.4.	39
Albumin nanoparticles	High pressure homogenization	Artemisinin	Human Serum Albumin	Developed nanoformulation with the lowest dimension that is feasible while maintaining outstanding uniformity and entrapment efficiency.	40
Lipid Nanoemulsions	High-pressure homogenization	Dihydroartemisinin	Soybean oil, Polyethylene glycol 4000	The formulations exhibited non-Newtonian flow and notable drug content efficiency within the 77-96% range. The formulations demonstrated effective parasitaemia clearance without causing cell hemolysis.	41
Surface-modified Nanostructured lipid carrier (NLC)	Hot homogenization	Dihydroartemisinin	Softisan®154, Tetracarpidium conophorum oil, PEG 4000	A 24-hour investigation on ex vivo permeation revealed sustained-release of DHA. For seven days, the rats' egg albumin-induced inflammation was reduced by the gels for a continuous period of eight hours. The creation of a gel with surface-modified lipid nanoparticles and DHA led to the controlled release of the medication to reduce localized inflammation.	42
Eudragit nanoparticles	Cold homogenization technique and nanoprecipitation technique	Silymarin	Eudragit RS 100 & Eudragit LS 100, PVA	Eudragit nanoparticles were risk-free and could have improved silymarin's pharmacological hepatoprotective abilities. Eudragit nanoparticles showed to be a helpful carrier when it comes to increasing the oral bioavailability of poorly soluble medicines.	43
Solid lipid nanoparticles	Cold homogenization	Myricitrin	Compritol, Oleic acid, Tween 80 and Span 20	In mouse and myotube cells, SLNs of myricitrin exhibited antiapoptotic, anti-diabetic, and antioxidant properties.	44
Solid lipid nanoparticles	High-pressure homogenisation	Curcumin	Sefsol-218®, Dynasan 114®	It proved potential to effectively construct C-SLNs with enhanced dispersibility and chemical stability in an aqueous solution. C-SLNs could be a helpful method for delivering curcumin, a cancer treatment.	45
Human serum albumin (HSA) Nanoparticles	coacervation	Noscapine	HSA, glutaraldehyde (Crosslinker)	Noscapine loaded HSA nanoparticles provided a viable method for specifically delivering drugs to tumor cells by increasing the medication's distribution and bioavailability and lowering the body's reaction to drug resistance.	46
Nanostructured lipid carriers (NLCs)	Hot high-pressure homogenisation	Docetaxel (DTX)	Compritol 888, Miglyol 812N, Soybean lecithin and Brij 78	NLCs demonstrated more consistent and effective DTX loading. Docetaxel concentration in the lungs was found to be considerably greater in rats treated with NLCs than in rats given docetaxel solution, In vitro release data demonstrated a sustained drug release activity.	47
Solid lipid nanoparticles	High speed homogenization	Curcuminoids	Poloxamer 188, Dioctyl sodium sulfosuccinate, stearic acid, Glyceryl monostearate	In vitro release tests revealed that curcuminoids were released from solid lipid nanoparticles for up to 12 hours and the entrapment efficiency of curcuminoids was found up to 70% (w/w).	48
Submicron-emulsion	High-pressure homogenization	Vincristine-oleic acid ion-pair complex (VCR-OA)	Soybean lecithin, Solutol HS15, Soybean oil	In comparison to VCR solution, the pharmacokinetic analysis of VCR-OA-SME revealed a longer mean residence duration and increased cytotoxicity on tumor cells.	49
Solid lipid nanoparticles	High-pressure hot homogenization	Paclitaxel	Chitosan and Hyaluronan	The results show that chitosan-HA-coated SLNs improved intrinsic chemotherapeutic actions by aiding in the targeting, cellular absorption, and time/dose-controlled distribution and release of PTX.	50

**Table 2:** Illustration of imaging techniques used for herbal nanoformulations

Nanoformulations	Imaging techniques	Herbal moiety	Inferences	Ref.
Pluronic (F127) nanoparticles	*In-vivo* fluorescence imaging	Curcumin	The developed NPs exhibited 6.5 times greater fluorescence intensity in brain tissue over pure curcumin. Furthermore, an in vitro test using Congo red, an indicator for Aβ plaques, demonstrated that encapsulated curcumin retains its capacity to adhere with Aβ plaques.	89
Berberine loaded liposomes	*In-vivo* imaging system	Berberine	Liposomes accumulated primarily within infarcted cardiac tissue (3 days following myocardial infarction). These findings demonstrate that liposomes can promote the local distribution of anti-inflammatory medicines like berberine into injured cardiac tissue.	90
*Acacia Senegal* derived gold nanoparticles	Computed tomography	*Acacia Senegal*	The findings suggest that naturally found Gum Arabic from *Acacia Senegal* may be utilized as a safe phytochemical component in the development of simply administrable biocompatible gold nanoparticles (AuNPs) for therapeutic and diagnostic uses in nanotechnology.	91
*Amaranthus spinosus* derived gold nanoparticles.	Molecular imaging, i.e. PET, MRI, SPECT	*Amaranthus spinosus*	AuNPs coated with *Amaranthus spinosus* leaf extract can be used for drug administration and molecular imaging techniques including MRI (magnetic resonance imaging), PET (positron emission tomography), and SPECT (single photon emission computerized tomography).	92
Fe3O4@*AstragalusPolysaccharide* Nanoparticles (Fe3O4@APS NPs)	Magnetic resonance imaging	*Astragalus* *Polysaccharide*	As an MRI contrast agent, Fe3O4@APS NPs provide considerable signal enhancement. The MRI scans of the major organs show no distinctive alterations even when being perfused for 16 hours, showing that NPs metabolism is safe.	93
Iron-polyphenol dendritic complexes	Magnetic resonance imaging	Green tea polyphenol	Both in vitro and in vivo, Fe^3+^-based polyphenol complexes suggested an effective T1 contrast impact and showed great magnetic resonance imaging influence for disease identification.	94
A Metal-Polyphenol Network Coated AuNR@MSN@MON (AMM)	Magnetic resonance imaging	Green tea polyphenol	The presence of Gd^3+^ improved magnetic resonance imaging (MRI) capabilities in the tumor milieu. The efficacy of this multipurpose nanotheranostic framework for addressing primary and metastatic carcinomas speaks well for future cancer therapy that brings together diagnostic and treatment.	95
*Cinnamomum zeylanicum* derived AuNPs	Near infra-red imaging	*Cinnamomum zeylanicum*	The synthesized Au nanoparticles exhibited photoluminescent properties, and the strength of photoemission increases with leaf broth content.	96
*Aloe barbadensis* derived iron oxide nanoparticles	Magnetic resonance imaging	*Aloe barbadensis*	At normal temperature, Fe_3_O_4_ nanoparticles exhibit superparamagnetic behavior, and the saturated magnetism of Fe_3_O_4_ nanoparticles improves with increased temperature of reaction and duration.	97

## Abbreviations

NDDS: Novel drug delivery system
SLNs: Solid Lipid Nanoparticles
HG: Hydrogels
RES: Reticuloendothelial system
HPO: Hydrogenated palm oil
CE: Carrot Extract
ME: Marigold Extract
ARF: Alkaloid-rich fraction
TNF-alpha: Tumour necrosis factor-alpha
IL: Interleukin
NLC: Nanostructured lipid carrier
RN: Rutin
IVIVC: *In vitro*-*in vivo* correlation
HPH: High pressure homogenization
SPE: Safflower petals extract
IVI: *In vivo* visual imaging
CPT: Camptothecin
CSNP: Polymeric hybrid nanoparticles
Cur: Curcumin
QD: Quantum dots
LSDs: Lysosomal storage disorders
IBD: Inflammatory bowel disease
AG: Andrographolide
CORM-2: Carbon monoxide donor
GA: Gambogic acid
QR: Quercetin
HSCs: Hepatic stellate cells
CD: Cyclodextrin
DSS: Dextran sulphate sodium
LPS: lipopolysaccharide
ROS: Reactive oxygen species
TNVs: Turmeric loaded nanovesicles
Fbs: fibroblasts
EGCG: Epigallocatechin-3-gallate
NCs: nanocages
HSCs: Hepatic stellate cells
DPP-TT: Diketopyrrolopyrrole-based conjugated polymer
SLB-HSA NCs: Silibinin albumin nanocrystals
RA: Rheumatoid arthritis
EMA: European Medicines Agency
HTA: Health Technology Assessment
